# αPIX Is a Trafficking Regulator that Balances Recycling and Degradation of the Epidermal Growth Factor Receptor

**DOI:** 10.1371/journal.pone.0132737

**Published:** 2015-07-15

**Authors:** Fanny Kortüm, Frederike Leonie Harms, Natascha Hennighausen, Georg Rosenberger

**Affiliations:** Institute of Human Genetics, University Medical Center Hamburg-Eppendorf, Hamburg, Germany; Loyola University Chicago, Stritch School of Medicine, UNITED STATES

## Abstract

Endosomal sorting is an essential control mechanism for signaling through the epidermal growth factor receptor (EGFR). We report here that the guanine nucleotide exchange factor αPIX, which modulates the activity of Rho-GTPases, is a potent bimodal regulator of EGFR trafficking. αPIX interacts with the E3 ubiquitin ligase c-Cbl, an enzyme that attaches ubiquitin to EGFR, thereby labelling this tyrosine kinase receptor for lysosomal degradation. We show that EGF stimulation induces αPIX::c-Cbl complex formation. Simultaneously, αPIX and c-Cbl protein levels decrease, which depends on both αPIX binding to c-Cbl and c-Cbl ubiquitin ligase activity. Through interaction αPIX sequesters c-Cbl from EGFR and this results in reduced EGFR ubiquitination and decreased EGFR degradation upon EGF treatment. However, quantitatively more decisive for cellular EGFR distribution than impaired EGFR degradation is a strong stimulating effect of αPIX on EGFR recycling to the cell surface. This function depends on the GIT binding domain of αPIX but not on interaction with c-Cbl or αPIX exchange activity. In summary, our data demonstrate a previously unappreciated function of αPIX as a strong promoter of EGFR recycling. We suggest that the novel recycling regulator αPIX and the degradation factor c-Cbl closely cooperate in the regulation of EGFR trafficking: uncomplexed αPIX and c-Cbl mediate a positive and a negative feedback on EGFR signaling, respectively; αPIX::c-Cbl complex formation, however, results in mutual inhibition, which may reflect a stable condition in the homeostasis of EGF-induced signal flow.

## Introduction

The identification of *ARHGEF6* encoding αPIX (also known as Cool-2; Entrez Gene ID: 9459) as a disease gene for a non-syndromic form of intellectual disability has brought this molecule into scientific focus [1]. αPIX belongs to the Dbl-related guanine nucleotide exchange factor (GEF) protein family [2, 3]. As a member of this molecule class, it specifically promotes the activity of the Rho-GTPases Rac1 and Cdc42 [3–8] by catalyzing the exchange of GDP for GTP within particular spatio-temporal contexts [9]. Rac1 and Cdc42 are key regulators of the actin cytoskeleton and affect diverse cellular processes, such as adhesion and migration, phagocytosis, cytokinesis, cell polarity, growth and cell survival, as well as neuronal morphogenesis [10–12]. In recent years αPIX turned out to regulate cell adhesion and motility [13–21], chemotaxis [22, 23], neuronal morphogenesis and function [8, 24, 25] as well as receptor-mediated signaling events [26–30].

The close homologue of αPIX, βPIX, has been identified as binding partner of Cbl proteins [31]. In the same study, ectopic expression of Cbl-b competitively inhibited binding of αPIX to PAK, an established αPIX binding partner; thus an interaction between αPIX and Cbl-b has been suggested [31]. Mammalian Cbl proteins include c-Cbl (Entrez Gene ID: 867), Cbl-b and Cbl-c; they are involved in the regulation of signal transduction in various cell types and in response to different stimuli. Cbl proteins are multifunctional adaptor proteins with ubiquitin ligase (E3) activity, thereby catalyzing ubiquitination of substrate proteins [32–34]. Modification with ubiquitin is classically associated with targeting proteins to proteasomes for degradation [35]. Moreover, ubiquitination has non-proteasomal functions during the internalization and postendocytic sorting of transmembrane proteins [36]. The role of Cbl as a negative regulator of receptor tyrosine kinase (RTK) signaling has been extensively studied [33, 37] and epidermal growth factor receptor (EGFR; Entrez Gene ID: 1956) has been the primary experimental model to examine the contribution of Cbl proteins to endocytic sorting of RTKs. Upon ligand binding, EGFR is rapidly internalized and sorted into endosomes; from there EGFR can be either recycled back to the cell surface or transported to lysosomes for degradation—a process called receptor downregulation [38]. Ubiquitination of EGFR by Cbl ubiquitin ligases has been implicated in ligand-mediated internalization/endocytosis and endosomal sorting of the EGFR [38, 39]. However, whereas ubiquitination seems to be dispensable for EGFR internalization, this modification strongly affects the postendocytic EGFR fate by lysosomal targeting and subsequent degradation of ubiquitinated receptors [38, 39].

Cbl action on EGFR ubiquitination and downregulation is negatively influenced by βPIX, and two possible mechanisms have been proposed. First, βPIX sequesters Cbl from EGFR, thereby preventing EGFR ubiquitination and downregulation [40, 41]; and second, βPIX, Cbl and EGFR form a stable complex at the plasma membrane, which blocks EGFR endocytosis, probably by preventing Cbl from engaging essential endocytic proteins [41, 42]. Obviously, both regulatory scenarios enable fine tuning of EGFR signaling; however, the remaining main question relates to the relative importance of the Cbl::PIX complexes in the regulation of specific endocytic sorting routes including internalization, degradation and recycling. Here we report on detailed analyses to determine the most relevant function of αPIX and c-Cbl in the control of EGFR endocytic pathways. We show that αPIX reduces EGFR degradation, most likely by αPIX-mediated sequestration of c-Cbl. However, in addition to this and quantitatively strongly prevailing, αPIX promotes EGFR recycling independently of c-Cbl binding. Together, our findings highlight an as yet unknown role for αPIX as a potent bimodal regulator of EGFR trafficking by controlling receptor recycling and degradation.

## Results

### αPIX interacts with c-Cbl

**Fig 1:**
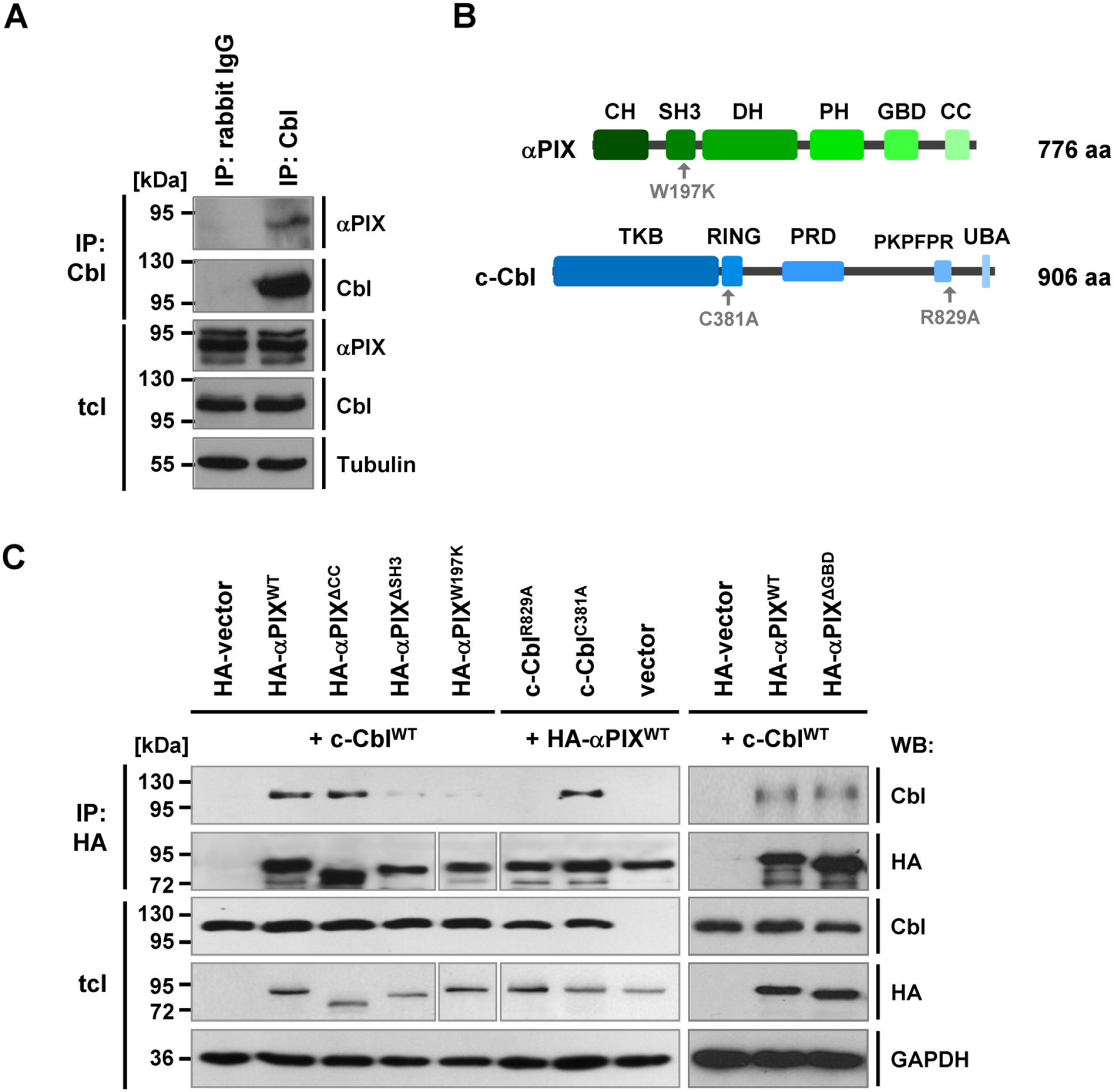
αPIX binds to the E3 ubiquitin ligase c-Cbl. A. Lysates from CHO-K1 cells were subjected to co-immunoprecipitation, either using rabbit IgG or rabbit anti-Cbl (S.C., Santa Cruz) antibodies. Total cell lysates (tcl) and immunoprecipitates (p) were resolved on an SDS-polyacrylamide gel and analyzed by immunoblotting using the indicated antibodies. B. Modular architecture of αPIX and c-Cbl. The protein domains of αPIX (CH, calponin homology; SH3, src-homology 3; DH, Dbl homology; PH, pleckstrin homology; GBD, GIT-binding domain; CC, coiled-coil domain) and c-Cbl (TKB, tyrosine-kinase-binding region; RING, RING finger domain; PRD, proline-rich domain; PKPFPR binding motif; UBA, ubiquitin associated domain) are schematically shown. Amino acid substitutions that are functionally important for this study are indicated. The total number of amino acids (aa) for αPIX and c-Cbl is given. C. Trp^197^ in αPIX and Arg^829^ in c-Cbl are essential for the αPIX::c-Cbl interaction. COS-7 cells were transfected with the indicated expression constructs. HA-tagged αPIX was immunoprecipitated from cell extracts by using anti-HA-conjugated agarose beads. After SDS-PAGE and western blotting, immunoprecipitates (IP) and total cell lysates (tcl) were probed with anti-HA and anti-Cbl antibodies. The HA-membrane was re-probed using anti-GAPDH antibodies to control for equal loading.

The interaction between ectopically expressed αPIX and c-Cbl has been previously shown by Seong et. al [21], whereas demonstration of an αPIX and Cbl association in a native environment was still pending. To determine whether endogenous αPIX and Cbl proteins also interact, we performed co-immunoprecipitation assays in CHO-K1 cells. We could co-precipitate αPIX of 87 kDa with c-Cbl indicating a specific protein-protein interaction in these cells when cultivated under basal (10% FBS) conditions (Fig 1A). It has been demonstrated that two tryptophan residues at position 196 and 197 within the SH3 domain of αPIX and two arginine residues in the PKPFPR motif of c-Cbl are crucial for this mutual interaction [21]. To verify αPIX::c-Cbl binding and to further delineate the structural requirements in αPIX and c-Cbl for their interaction (Fig 1B), we ectopically expressed different protein variants in COS-7 cells and performed co-immunoprecipitation experiments. Overexpressed wild-type αPIX (αPIX^WT^) strongly co-precipitated with overexpressed wild-type c-Cbl (c-Cbl^WT^) (Fig 1C). Both deletion of the SH3 domain (αPIX^ΔSH3^) as well as substitution of the conserved tryptophan at position 197 within the SH3 domain of αPIX (αPIX^W197K^) resulted in drastically diminished co-immunoprecipitation of wild-type c-Cbl (c-Cbl^WT^) (Fig 1C). In contrast, deletion of the GIT-binding domain (αPIX^ΔGBD^) did not impair interaction with c-Cbl (Fig 1C). This motif enables binding of αPIX to the multifunctional GIT (G protein-coupled receptor kinase-interacting target) family proteins (S1 Fig) [43, 44]. αPIX lacking the coiled-coil domain (αPIX^ΔCC^) which is assumed to enable dimerization and trimerization of αPIX and βPIX molecules [6, 17, 45] still co-precipitated c-Cbl (Fig 1C). Importantly, substitution of arginine 829 by alanine in the PKPFPR motif of c-Cbl (c-Cbl^R829A^) abolished interaction with αPIX^WT^ indicating that both αPIX and βPIX compete for the same c-Cbl binding motif (Fig 1C). However, the E3 ligase activity-deficient c-Cbl^C381A^ mutant [46] still co-precipitated with αPIX (Fig 1C), suggesting that a functional c-Cbl RING finger motif which facilitates protein ubiquitination is not essential for αPIX::c-Cbl complex formation.

Taken together, our data strongly support a specific interaction of αPIX and c-Cbl.

### EGF stimulation controls αPIX::c-Cbl complex formation which in turn is required for EGF-induced αPIX and Cbl protein turnover

**Fig 2:**
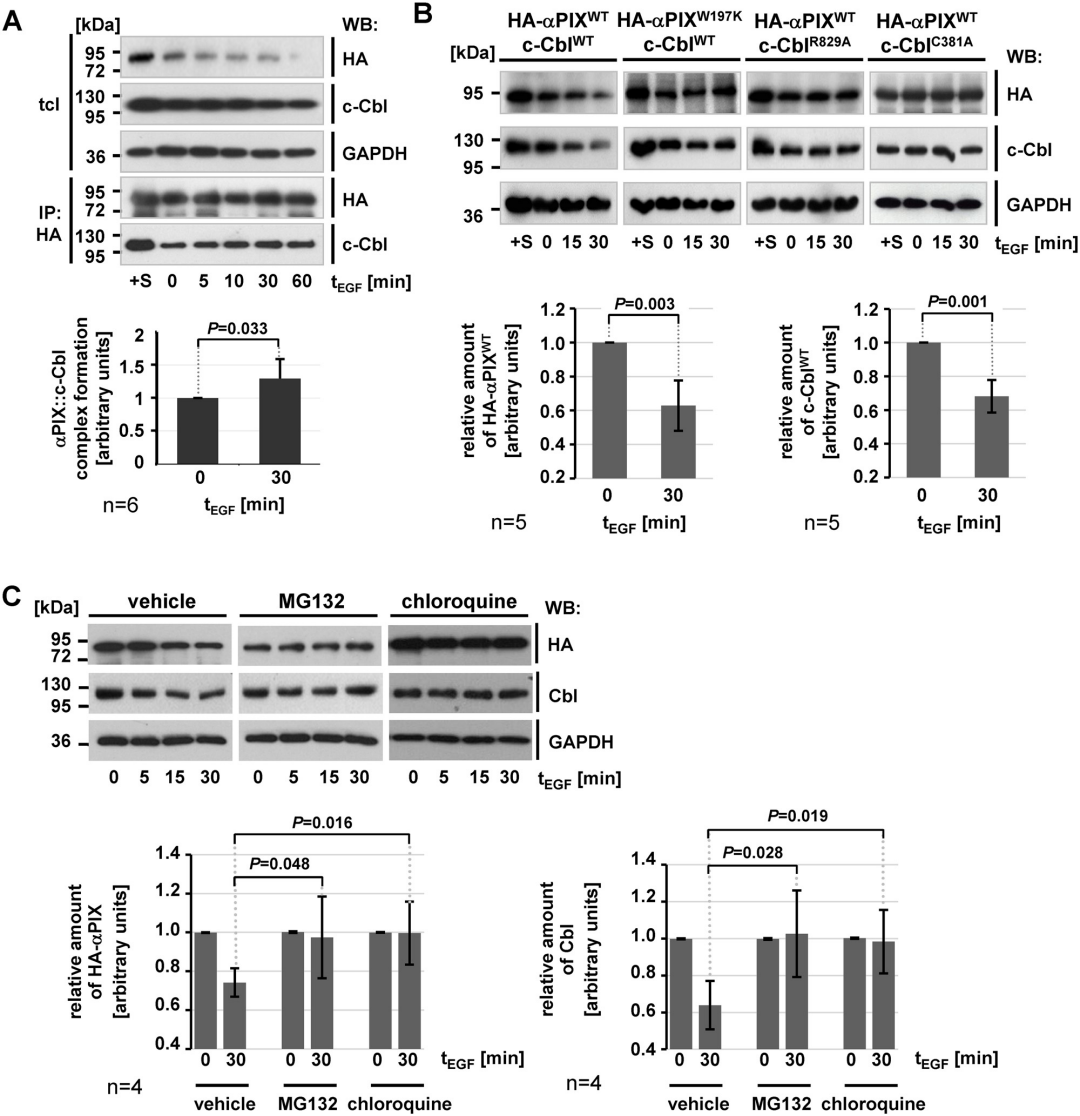
αPIX::c-Cbl complex formation and degradation. A. EGF regulates complex formation of αPIX and c-Cbl. COS-7 cells transiently co-expressing HA-αPIX^WT^ and c-Cbl^WT^ were serum-starved or cultured under basal growth conditions (+S). Starved cells were stimulated with 5 ng/ml EGF for 5, 10, 30 or 60 min at 37°C (t_EGF_) or left untreated (0 min). αPIX was immunoprecipitated from cell extracts by using anti-HA antibodies and protein levels of HA-αPIX, c-Cbl and GAPDH were determined in cell lysates (tcl) and precipitates (IP) by immunoblotting. Based on densitometric quantification of autoradiographic signals derived from immunoblots, the graphs show relative amounts of c-Cbl co-precipitated with HA-αPIX in unstimulated cells and at 30 min upon EGF induction. Amounts of c-Cbl in the precipitates were normalized to total c-Cbl and considered as 1 for unstimulated cells (0 min). Data represent the mean of six (n = 6) independent experiments ± sd. *P* value was calculated by paired Student’s t-test. B. Downregulation of αPIX and c-Cbl depends on complex formation of these proteins. COS-7 cells transiently expressing various HA-αPIX and c-Cbl protein variants were cultivated under basal growth conditions (+S), serum starved (0 min) or serum-starved and stimulated with 5 ng/ml EGF for 15 or 30 min. Cells were harvested and protein levels of HA-αPIX, c-Cbl, and GAPDH were determined by immunoblotting. Based on densitometric quantification of autoradiographic signals derived from immunoblots, the graphs show relative amounts of HA-αPIX^WT^ and c-Cbl^WT^ in the total lysates from cells overexpressing HA-αPIX^WT^ and c-Cbl^WT^. Measurements were normalized to GAPDH and considered as 1 for unstimulated cells (0 min t_EGF_). Data represent the mean of five (n = 5) independent experiments ± sd. *P* values were calculated by paired Student’s t-test. C. Both proteasomal and lysosomal inhibitors prevent EGF-induced αPIX and c-Cbl degradation. Serum-starved COS-7 cells transiently co-expressing HA-αPIX^WT^ and c-Cbl^WT^ were incubated with 20 μM MG132 or 50 μM chloroquine for 6h or left untreated (vehicle). Upon stimulation with 25 ng/ml EGF for the indicated times, cell extracts were subjected to SDS-PAGE and immunoblotting using anti-HA and anti-Cbl antibodies. Blots were reprobed with anti-GAPDH antibody to test for loading equality. Based on densitometric quantification of autoradiographic signals derived from immunoblots, the graphs show relative amounts of HA-αPIX and Cbl in the cell lysates. Measurements were normalized to GAPDH and considered as 1 for unstimulated cells (0 min t_EGF_). Data represent the mean of four (n = 4) independent experiments ± sd. *P* values were calculated by unpaired Student’s t-test.

The implication of c-Cbl in EGF-induced EGFR downregulation [38, 39] raises the question whether EGF stimulation regulates αPIX::c-Cbl complex formation and/or αPIX and c-Cbl protein turnover. To test this, we transiently co-expressed HA-αPIX and c-Cbl wild type in COS-7 cells and immunoprecipitated αPIX from cell lysates at various times after EGF stimulation following serum starvation. We noticed that both ectopically expressed αPIX and c-Cbl protein levels decreased over time in total cell lysates (Fig 2A, 1^st^ and 2^nd^ panel). In contrast, in the precipitates we observed a gradual increase of co-precipitated c-Cbl until 30 min of EGF stimulation (Fig 2A, bottom panel; for quantification see graph in Fig 2A). Interestingly, the strongest signal for c-Cbl in the precipitates was found in cells cultured under saturated conditions (+10% FBS), whereas upon serum-starvation little c-Cbl co-precipitated with αPIX (Fig 2A, bottom panel, 1^st^ and 2^nd^ lane). Since immunodepletion of the primary antigen (HA-αPIX) was not complete in this assay, the amounts of HA-αPIX in the precipitates (Fig 2A, 4^th^ panel) were similar and signals for co-precipitated c-Cbl (Fig 2A, bottom panel) could be directly compared. Thus, EGF (or FBS) abundance seems to stabilize the αPIX::c-Cbl interaction, thereby increasing the number of αPIX::c-Cbl complexes in relation to uncomplexed αPIX and c-Cbl molecules. Our data suggest that αPIX preferentially binds to c-Cbl in the late phase of EGF stimulation and under saturated growth conditions, whereas αPIX and c-Cbl are mainly uncomplexed in growth factor- or EGF-starved cells and during the early phase of EGF stimulation. Next, we specified molecular determinants for EGF-induced αPIX and c-Cbl downregulation. αPIX and c-Cbl decrease depends on their interaction as expression of the binding-deficient variants αPIX^W197K^ or c-Cbl^R829A^ in COS-7 cells stabilized αPIX and c-Cbl protein levels upon EGF stimulation (Fig 2B). Moreover, co-expression of αPIX with the E3 ligase activity-deficient c-Cbl^C381A^ mutant abolished EGF-induced decrease of αPIX and c-Cbl protein amounts (Fig 2B). This indicates that αPIX::c-Cbl complex formation and a functional c-Cbl RING domain are prerequisites for EGF-induced degradation of αPIX and c-Cbl. We examined which degradative system could be responsible for EGF-induced decrease of αPIX and c-Cbl levels. Proteasomal inhibition by MG132 maintained αPIX and c-Cbl protein amounts (Fig 2C), suggesting that subsequent to EGF stimulation αPIX and c-Cbl enter the proteasomal degradation pathway. However, inhibition of lysosomal degradation by using chloroquine also stabilized αPIX and c-Cbl protein levels (Fig 2C). These data do not allow to define a specific pathway for the degradation of αPIX and c-Cbl.

### αPIX regulates trafficking of EGFR

**Fig 3:**
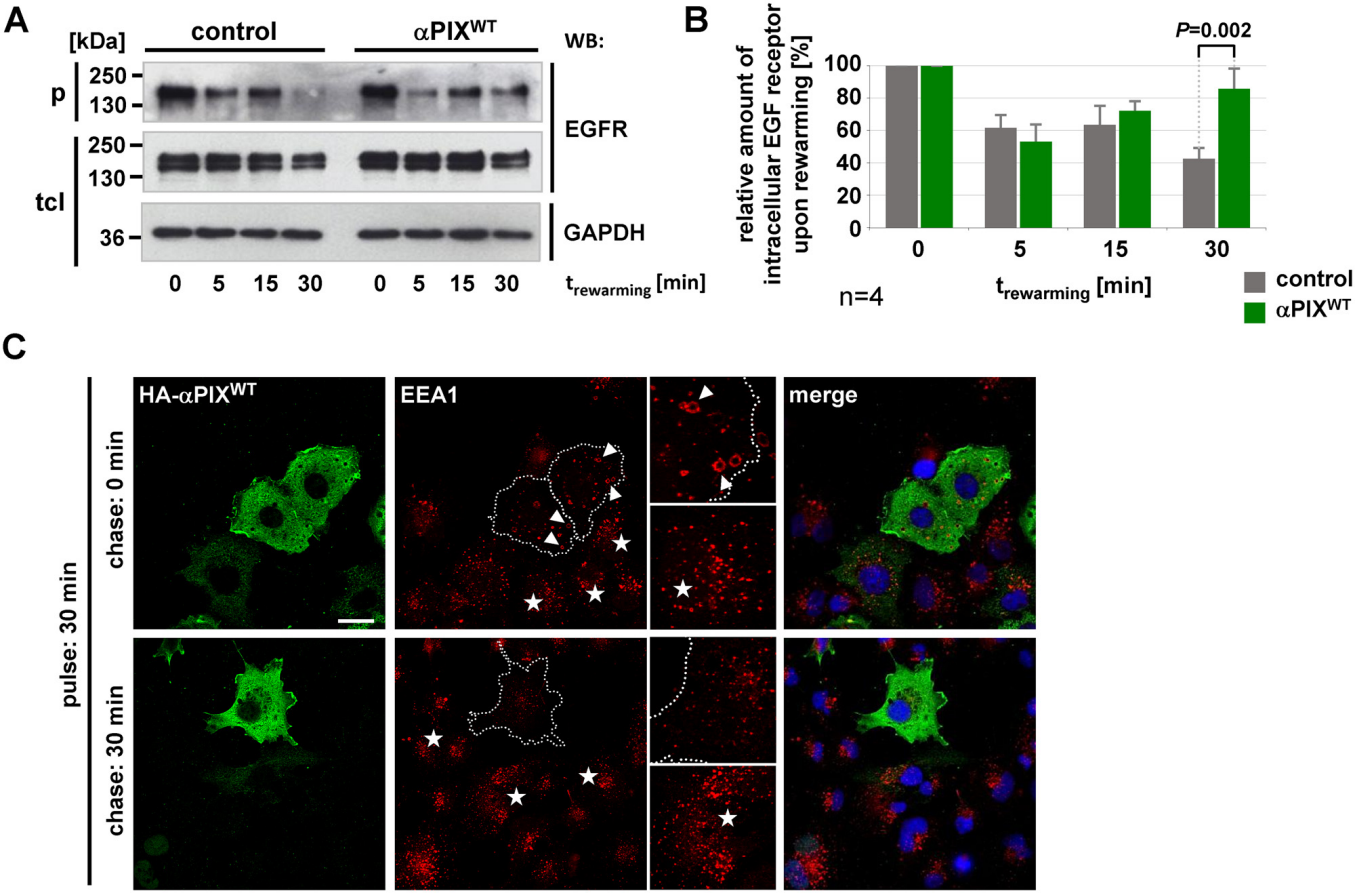
αPIX regulates EGFR trafficking. A. Stable αPIX^WT^ and control (CAT) CHO cell lines were transiently transfected with EGFR expression constructs. Following serum starvation, surface proteins were biotinylated and cells were stimulated with 25 ng/ml EGF for 30 min at 37°C (pulse) to induce EGF receptor trafficking. Then, cells were transferred to 4°C, residual surface biotin was removed and cells were rewarmed to 37°C for the indicated times (chase). Recycled surface proteins were de-biotinylated and intracellular biotinylated proteins were precipitated from cell extracts. Parallel cultures were harvested before rewarming (0 min). Representative autoradiographs show EGFR levels in precipitates (p) and total cell lysates (tcl). Equal loading was verified by reprobing membranes with anti-GAPDH antibodies. B. Based on densitometric quantification of autoradiographic signals derived from EGFR trafficking assays (A), the graphs show relative amounts of intracellular EGFR. Precipitated (intracellular) EGFR fractions were normalized to total EGFR levels and considered as 100% in cultures that haven’t been rewarmed (0 min). Data represent the mean of four (n = 4) independent experiments ± sd. *P* values were calculated by unpaired Student’s t-test. C. Serum-starved COS-7 cells transiently expressing HA-αPIX^WT^ were stimulated with 25 ng/ml EGF for 30 min (pulse). Subsequently, cells were either immediately fixed (0 min) or incubated in starvation medium for further 30 min (chase) and then fixed. HA-tagged αPIX was visualized by staining with anti-HA followed by Alexa Fluor 488-conjugated secondary antibodies. The early endosomal compartment was visualized by anti-EEA1 antibodies followed by Alexa Fluor546-conjugated antibodies and the nucleus was detected by staining with DAPI. Note the enlarged morphology (arrowheads, upper panel) and the reduced number (outlined cell, lower panel) of EEA1-positive vesicles in αPIX overexpressing cells compared to surrounding non-transfected cells (asterisks) at 0 min and 30 min chase, respectively. Specific details are shown enlarged at the right hand side of the images. 50 cells each (non-transfected cells and HA-αPIX^WT^ overexpressing cells) derived from three independent specimen have been examined, representative cells are shown. Scale bar, 20 μm.

Due to the prominent function of c-Cbl in the regulation of EGFR degradation [38], we checked if αPIX is part of the EGFR signaling pathway by controlling endocytic traffic of EGFR. For investigation of trafficking of ectopically expressed EGFRs, CHO cells have proven to be a good model system, because they express few endogenous EGFRs but contain all of the appropriate machinery for endocytic EGFR traffic [47]. Therefore, we established CHO cell lines stably expressing V5-tagged αPIX^WT^, αPIX^W197K^, the exchange activity deficient variant αPIX^GEF-^ (for details see Materials and Methods), GIT binding deficient αPIX^ΔGBD^, or chloramphenicol acetyl transferase (CAT) as control. Stable expression of transgenes was demonstrated by immunoblotting (S2 Fig). To quantitatively analyze the influence of αPIX on EGFR turnover, we used surface biotinylation-based pulse-chase assays modified after well-established protocols [48–52], thereby looking at synchronized waves of EGFR trafficking. In a first approach, we followed the amount of intracellular EGFR at various times (chase) subsequent to 30 min EGF stimulation (pulse) and removal of EGF in CHO cell lines stably expressing αPIX or CAT (control). At time 0 min the EGFR level in the precipitates corresponded to the amount of biotinylated intracellular EGFR that is available for trafficking; this EGFR fraction was very similar in αPIX^WT^ expressing and control cells (Fig 3A). After 5 min rewarming, i.e. when EGFR trafficking resumed, we observed only little intracellular EGFR suggesting that EGFR has been either degraded or recycled to the membrane in both cell lines (Fig 3A and 3B). From 5 to 15 min the intracellular EGFR fractions increased in αPIX^WT^ and control cells with similar rates indicating efficient re-internalization of recycled EGFR. However, after 30 min rewarming, EGFR accumulated in αPIX^WT^ expressing cells and not in control cells (Fig 3A and 3B). This enrichment of intracellular EGFR in αPIX^WT^ cells could be explained by either reduced EGFR degradation or increased EGFR recycling to the cell surface and re-internalization. We could strengthen our hypothesis of a functional involvement of αPIX on intracellular trafficking processes by microscopic examination of immunofluorescently stained COS-7 cells. This cell type expresses high levels of endogenous EGFR and, thereby, is typically used for detection of trafficking processes by fluorescent microscopic methods [47]. Transient expression of αPIX^WT^ resulted in fewer but enlarged EEA1-positive vesicular structures (Fig 3C, upper panel, arrowheads) compared with untransfected cells (Fig 3C, upper panel, asterisks) after an EGF pulse (0 min). Upon a chase phase of 30 min, the number of EEA1-stained vesicles was markedly reduced in αPIX^WT^ overexpressing cells (Fig 3C, lower panel, dashed line) compared to the surrounding non-transfected cells (Fig 3C, lower panel, asterisks). These results indicate that αPIX interferes with the turnover/trafficking of early endosomes.

The following experiments aimed to differentiate between the effects of αPIX^WT^ expression on EGFR degradation and EGFR recycling/re-internalisation.

### αPIX limits EGFR degradation by sequestration of c-Cbl

**Fig 4:**
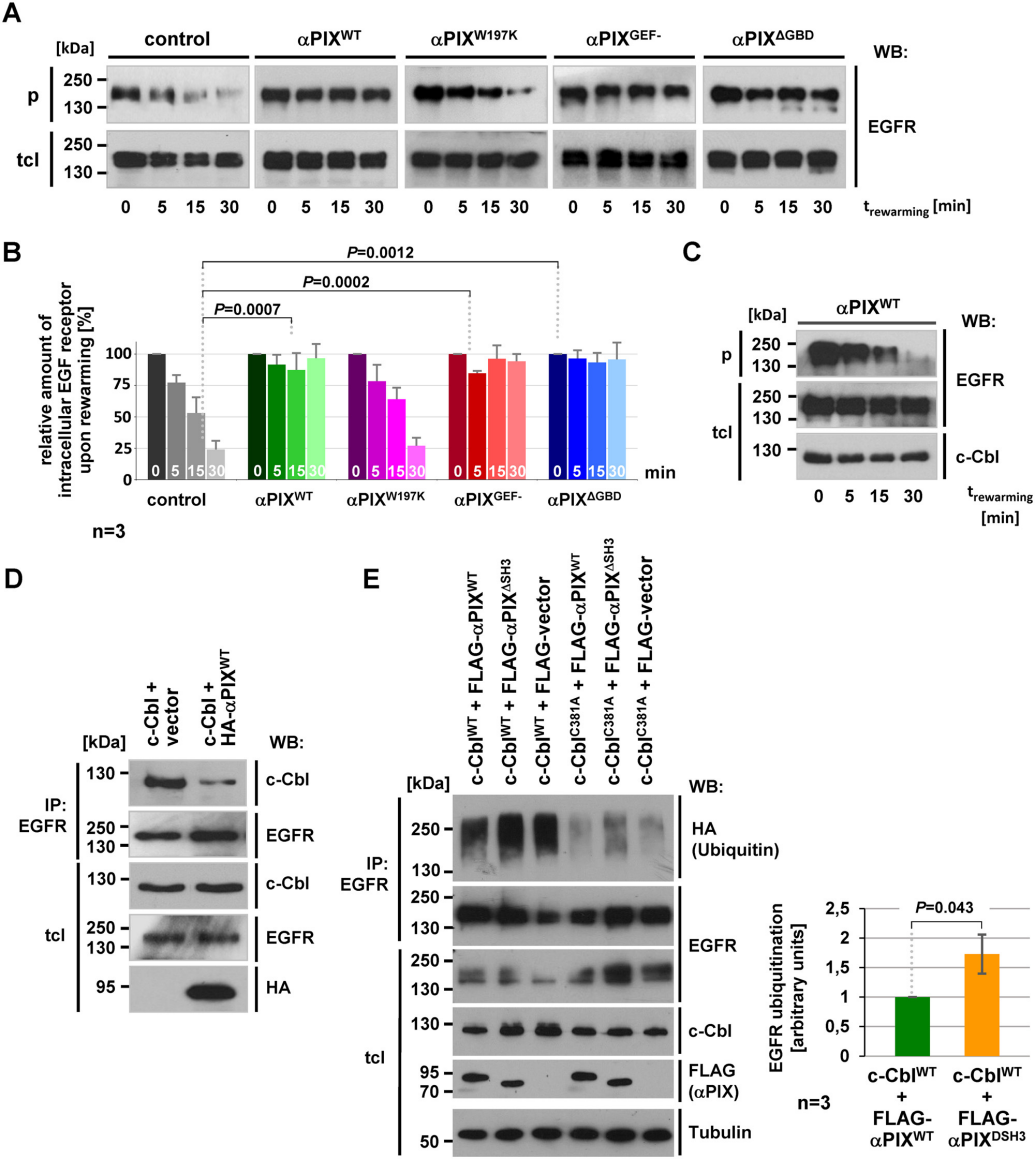
αPIX interferes with EGFR ubiquitination and degradation. A. CHO cells stably expressing the indicated αPIX protein variants or CAT (control) were transfected with an EGFR expression construct. The experimental procedure was essentially the same as described in Fig 3A, except for the medium that was supplemented with 0.3 mM of the recycling inhibitor primaquine to block EGFR recycling. Cells were harvested after various times of rewarming followed by surface de-biotinylation (5, 15, 30 min) or before rewarming (0 min). Intracellular biotinylated proteins were precipitated from cell extracts and cell lysates (tcl) and precipitates (p) were subjected to immunoblotting using anti-EGFR antibodies. B. Graphs show relative amounts of intracellular EGFR derived from densitometric quantification of autoradiographic signals obtained from EGFR degradation assays (A). Precipitated (intracellular) EGFR fractions were normalized to total EGFR levels and considered as 100% in cultures that haven’t been rewarmed (0 min). Data represent the mean of three (n = 3) independent experiments ± sd. *P* values were calculated by unpaired Student’s t-test. C. c-Cbl co-expression rescues αPIX^WT^-induced inhibition of EGFR degradation. CHO cells stably expressing αPIX^WT^ were co-transfected with EGFR and c-Cbl expression constructs and subsequently treated as described in Fig 3A. After immunoblotting cell lysates (tcl) were probed with anti c-Cbl and anti-EGFR antibodies, and precipitates (p) were probed with anti-EGFR antibodies. D. αPIX^WT^ sequesters c-Cbl from EGF receptors. COS-7 cells were transfected with expression constructs as indicated. Endogenous EGFR was immunoprecipitated from cell extracts by using anti-EGFR antibodies. Upon SDS-PAGE and western blotting, precipitates (IP) and total cell lysates (tcl) were probed with anti-EGFR and anti-Cbl antibodies. Expression of αPIX^WT^ was demonstrated by immunodetection with anti-HA antibodies. E. αPIX^WT^ reduces c-Cbl-mediated EGFR ubiquitination. COS-7 cells were transiently co-transfected with *c-Cbl* and FLAG-tagged *αPIX* expression constructs (as indicated) together with HA-tagged ubiquitin and EGFR expression constructs. For control purpose cells were transfected with empty FLAG-vector. Subsequent to incubation under serum-free culture conditions overnight, cells were stimulated with 20 ng/ml EGF for 30 min and harvested. EGFR was immunoprecipitated with anti-EGFR antibodies and protein A-agarose and samples were subjected to SDS-PAGE and immunoblotting. Levels of ubiquitinated EGFR in precipitates (IP) were monitored by using anti-HA antibodies. EGFR levels in total cell lysates (tcl) and precipitates (IP) were determined by using anti-EGFR antibodies and expression of c-Cbl and FLAG-αPIX protein variants in total cell lysates was shown by using anti-c-Cbl and anti-FLAG antibodies, respectively. Tubulin served as a loading control. Representative blots from one out of three independent experiments are shown. Based on densitometric quantification of autoradiographic signals derived from immunoblots, the graph shows relative amounts of ubiquitinated EGFR. Amounts of HA-ubiquitinated EGFR in the precipitates were normalized to total EGFR and considered as 1 for cells expressing FLAG-αPIX^WT^. Data represent the mean of three (n = 3) independent experiments ± sd. *P* value was calculated by paired Student’s t-test.

To focus on receptor degradation we surface-biotinylated CHO cells stably expressing various αPIX protein variants, treated cultures with EGF for 30 min (pulse) in the presence of the recycling inhibitor primaquine [53], and subsequently chased in primaquine-containing medium for 5, 15 and 30 min at 37°C. With this procedure we minimized the contribution of recycling and re-internalization to the amount of intracellular EGFR receptors. In control cells intracellular EGFR levels constantly decreased over time indicating that the internalized EGFR is subject to degradation (Fig 4A and 4B). In contrast, intracellular EGFR remained constant over the observation period in αPIX^WT^ cells suggesting that αPIX negatively influences degradation of EGFR (Fig 4A and 4B). This effect does not depend on αPIX GEF activity and its GIT binding domain as expression of αPIX^GEF-^ and αPIX^ΔGBD^ resulted in constant intracellular EGFR levels (Fig 4A and 4B). However, substitution of the c-Cbl binding-critical amino acid tryptophan 197 for lysine (αPIX^W197K^) reversed this effect (Fig 4A and 4B). These data demonstrate that αPIX negatively influences EGFR degradation and give rise to speculate that binding with c-Cbl underlies this function. Indeed, inhibition of EGF-induced EGFR degradation in αPIX^WT^ cells was rescued by coexpression of c-Cbl (Fig 4C) indicating that this effect is not mediated by other αPIX interacting proteins, such as p21 activated kinases which also bind to the SH3 domain of αPIX [2, 3].

It has been proposed that βPIX sequesters Cbl from EGFR resulting in reduced EGFR degradation [40, 41]. To test if αPIX also interferes with complex formation between c-Cbl and EGFR, we performed co-immunoprecipitation experiments. We could co-immunoprecipitate ectopically expressed c-Cbl with endogenous EGFR from COS-7 cells; however, when αPIX was co-expressed, c-Cbl co-precipitation with EGFR was strongly impaired (Fig 4D).

To determine whether c-Cbl mediated EGFR ubiquitination is influenced by αPIX we stimulated COS-7 cells with EGF for 30 min to induce receptor ubiquitination and, subsequently, performed precipitation-based EGFR ubiquitination assays. Overexpression of c-Cbl^WT^ resulted in a strong ubiquitination signal in EGFR precipitates (Fig 4E, 3^rd^ lane). Co-expression of wild-type αPIX but not of c-Cbl binding deficient αPIX^ΔSH3^ clearly decreased the level of EGFR ubiquitination (Fig 4E, 1^st^ and 2^nd^ lane). We conclude, that αPIX reduces c-Cbl depending EGFR ubiquitination. For control purpose we overexpressed ubiquitination-deficient c-Cbl^C381A^, which resulted in very low levels of ubiquitinated EGFR most likely representing receptors that have been ubiquitinated by endogenous ubiquitin ligases (Fig 4E, 4^th^-6^th^ lane).

Together, these data provide a straight forward explanation for the negative effect of αPIX on EGFR degradation: αPIX sequesters c-Cbl from EGFR which results in diminished EGFR ubiquitination and degradation.

### αPIX increases EGFR recycling mediated by its GIT binding domain (GBD)

**Fig 5:**
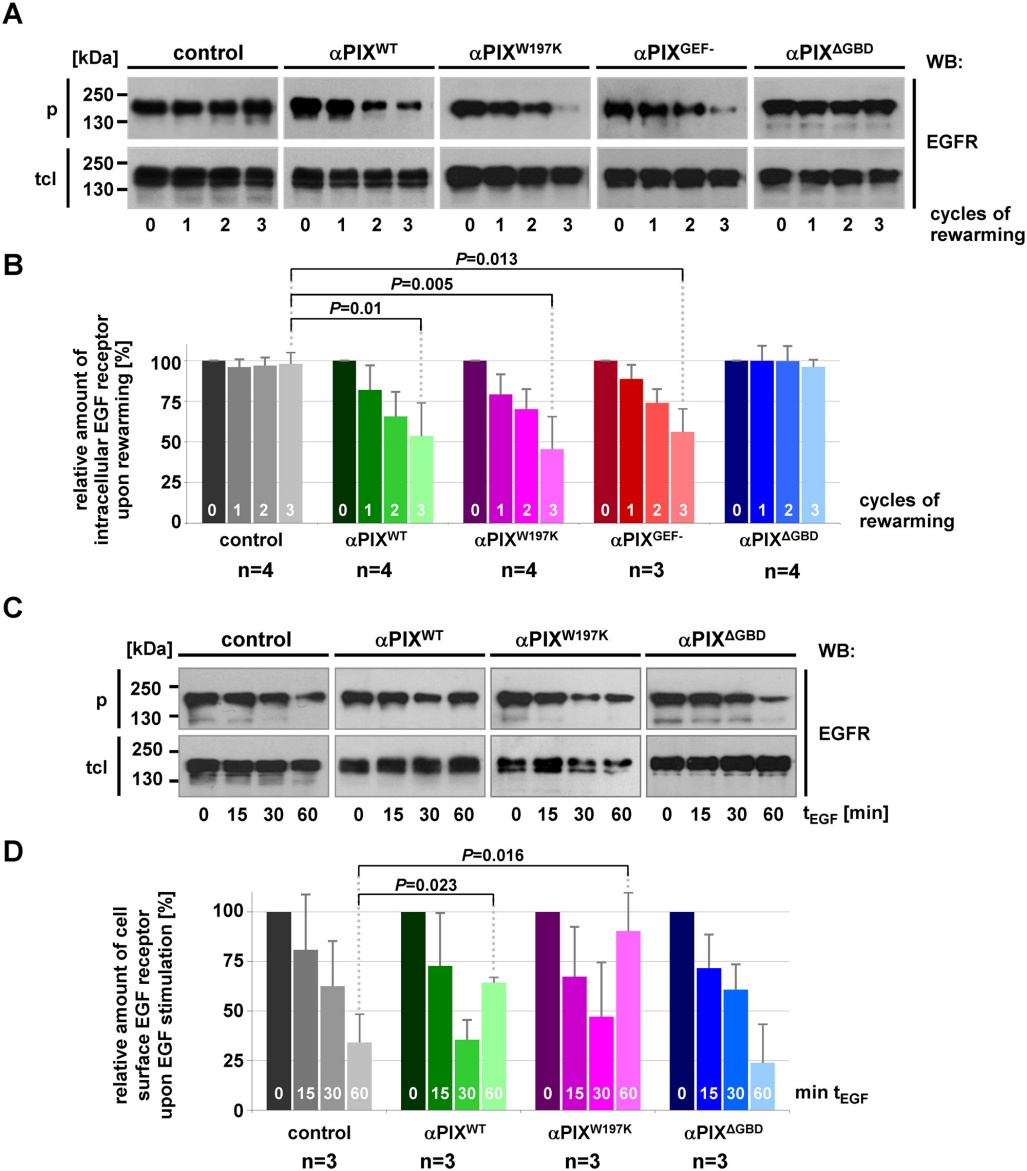
αPIX stimulates EGFR recycling. A. Pulse-chase settings: CHO cells stably expressing the indicated αPIX protein variants or CAT (control) were transfected with an EGFR expression construct followed by incubation in starvation medium supplemented with pepstatin A and leupeptin to inhibit lysosomal degradation. Surface proteins were biotinylated and cells were stimulated with 25 ng/ml EGF for 30 min at 37°C to induce EGF receptor trafficking. Subsequently, cells were transferred to 4°C and residual surface biotin was removed. Parallel cultures were subjected to 1, 2 or 3 cycles of 2 min rewarming at 37°C and de-biotinylation of recycled receptors. Intracellular biotinylated proteins were precipitated from cell extracts. Parallel cultures were harvested without rewarming/de-biotinylation (0 cycles). Total cell lysates (tcl) and precipitates (p) were subjected to SDS-PAGE and immunoblotting using anti-EGFR antibodies. Representative autoradiographs show EGFR levels. B. Graphs represent quantified densities of autoradiographic signals from EGFR recycling assays (A). Amounts of precipitated EGFR fractions were normalized to total EGFR levels and considered as 100% for parallel cultures that haven’t been rewarmed. Data represent the mean of four (control, αPIX^WT^, αPIX^W197K^, αPIX^ΔGBD^) or three (αPIX^GEF-^) independent experiments ± sd. *P* values were calculated by unpaired Student’s t-test. C. Steady state setting: CHO cells stably expressing the indicated αPIX protein variants or CAT (control) were transfected with EGFR expression constructs. Following serum starvation, cells were stimulated with 25 ng/ml EGF for 15, 30 or 60 min at 37°C or left unstimulated (0 min) and subsequently transferred to 4°C. Cell surface proteins were biotinylated on ice, precipitated from cell extracts and both cell lysates (tcl) and precipitates (p) were subjected to SDS-PAGE and immunoblotting using anti-EGFR antibodies. D. Graphs represent quantified densities of autoradiographic signals obtained from experiments as described in (C). Amounts of precipitated EGFR were normalized to total EGFR levels and considered as 100% for unstimulated parallel cultures. Data represent the mean of three independent experiments ± sd. *P* values were calculated by unpaired Student’s t-test.

Next, we performed recycling assays to analyze if αPIX also contributes to the transport of endocytozed EGFR back to the plasma membrane. We treated cells with leupeptin and pepstatin A for 24 h to inhibit lysosomal degradation, which allowed us to exclusively study EGFR recycling. After receptor biotinylation, we treated cells for 30 min with EGF (pulse) and then chased the fraction of internal EGFR. For this, parallel cell cultures were subjected to three rounds of recycling (2 min each) and removal of surface biotin; thereby, the amount of intracellular EGFR should gradually decrease due to progressive EGFR recycling. We observed a fast decrease of the intracellular EGFR pool over time in αPIX^WT^ expressing cells, whereas this fraction was not reduced in control cells (Fig 5A and 5B). Expression of αPIX^GEF-^ or αPIX^W197K^ similarly resulted in strong reduction of intracellular EGFR, which was not the case for αPIX^ΔGBD^ expressing cells (Fig 5A and 5B). These results demonstrate that αPIX strongly promotes post-endocytic sorting of EGFR to the cell surface, and this function depends on the αPIX GBD domain. We could substantiate a functional cooperation of αPIX and GIT proteins during EGFR recycling, because ectopic co-expression of GIT2 in αPIX^ΔGBD^ cells rescued the stimulatory effect of αPIX on recycling, suggesting a function of GIT2 downstream of αPIX (S3 Fig).

The pulse-chase experiments described above reflect an unphysiological situation. Under physiological conditions (steady state) of the continuous presence of EGF [54, 55], recycled receptors repeatedly undergo endocytosis resulting in a constant decrease of cell surface EGFR over time [50]. To examine the impact of αPIX on EGFR homeostasis under steady state conditions, we followed the amount of surface EGFR in cells stimulated with EGF for different time periods (without removing EGF). Both control and αPIX^WT^-expressing cells showed high levels of EGFR on the surface without EGF stimulation (Fig 5C and 5D; 0 min), which was followed by a decrease after 15 to 30 min of EGF stimulation. Thus, removal of EGFR from the cell surface until 30 min of EGF stimulation was comparable in αPIX^WT^ and control cells and we concluded that αPIX does not affect EGFR internalization. In control cells the amount of EGFR at the cell surface was further reduced until 60 min of EGF induction, whereas this EGFR fraction was restored in αPIX^WT^-expressing cells (Fig 5C and 5D). The kinetics of surface localization of EGFR in αPIX^WT^ cells was comparable to cells expressing αPIX^W197K^ but not to the αPIX^ΔGBD^ cell line which responded to EGF stimulation similar to control cells (Fig 5C and 5D). Our finding of a marked elevation of surface EGFR after 60 min of EGF treatment in αPIX^WT^-expressing cells confirms a stimulative effect of αPIX on EGFR recycling. The failure of αPIX^ΔGBD^ expressing cells to accumulate EGFR at the cell surface 60 min after EGF stimulation further underscores the importance of the GIT binding domain for this effect. Since αPIX^W197K^ also enhanced EGFR transport to the membrane we conclude that this function does not depend on the αPIX::c-Cbl interaction; thus, increased EGFR recycling is not a secondary effect of the inhibition of EGFR degradation by αPIX-mediated c-Cbl sequestration.

### Stimulation of EGFR recycling is the major function of αPIX

**Fig 6:**
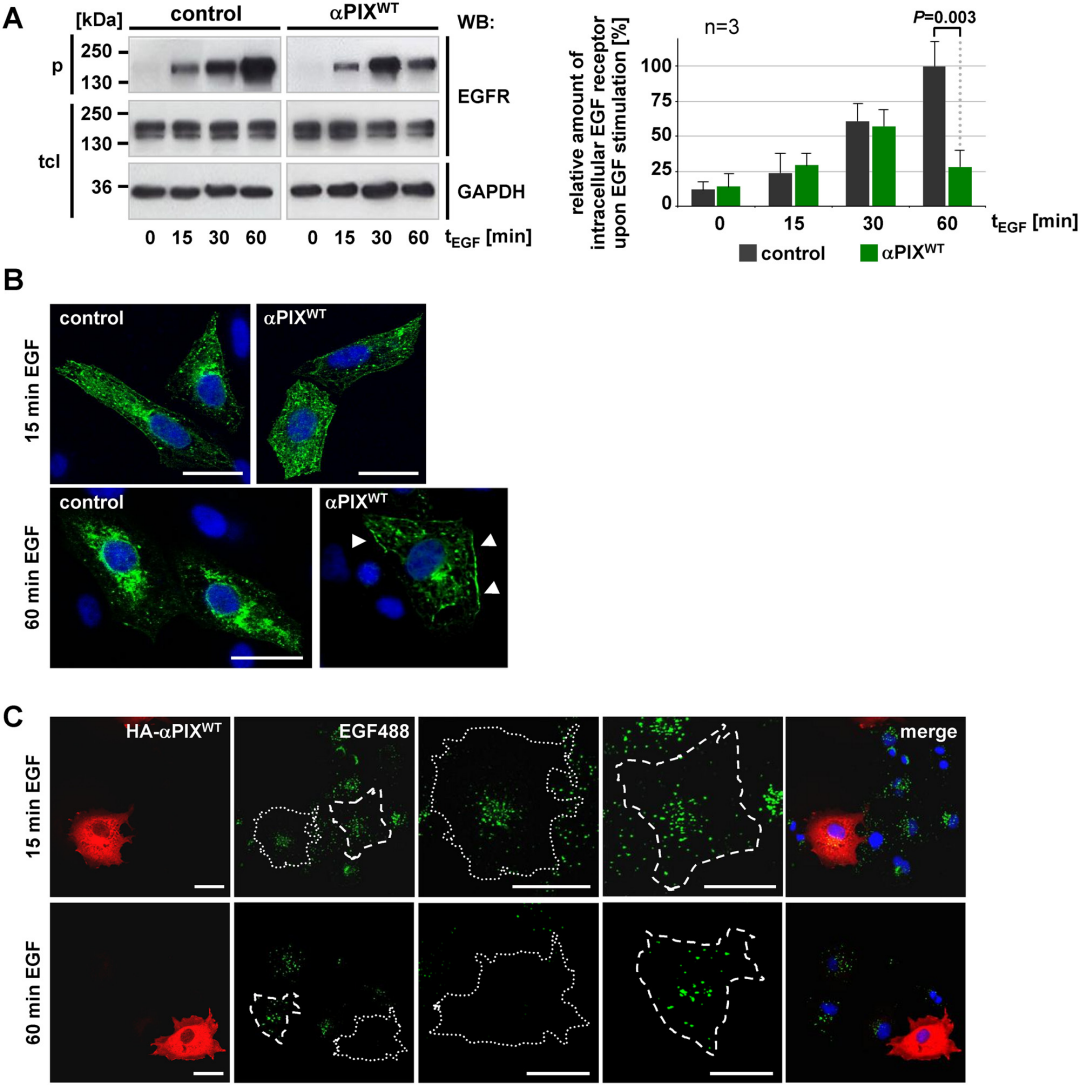
Stimulation of EGFR recycling is the dominant αPIX function during EGFR trafficking. A. CHO cells stably expressing αPIX^WT^ or CAT (control) were transfected with EGFR expression constructs. Following serum starvation overnight, surface proteins were biotinylated on ice and cells were stimulated with 25 ng/ml EGF for 15, 30 or 60 min at 37°C to induce EGF receptor trafficking. A parallel culture was left unstimulated (0 min). Cells were transferred to 4°C, surface proteins were de-biotinylated and intracellular biotinylated proteins were precipitated from cell extracts. Representative autoradiographs show EGFR levels in total cell lysates (tcl) and precipitates (p) upon SDS-PAGE and immunoblotting. GAPDH served as a loading control. Based on densitometric quantification of autoradiographic signals the graphs show relative amounts of intracellular EGFR. Amounts of precipitated EGFR were normalized to total EGFR levels and considered as 100% in control cells after 60 min EGF stimulation (note: standard deviation for control cells at 60 min t_EGF_ was calculated subsequent to normalization to total EGFR levels). Data represent the mean of three independent experiments ± sd. For *P* value was calculated by paired Student’s t-test. B. Immunocytochemical analysis of EGFR distribution. Stable αPIX^WT^ and control (CAT) CHO cells were transfected with EGFR constructs and serum-starved overnight. Cells were stimulated with 25 ng/ml EGF for 15 or 60 min at 37°C to induce EGF receptor trafficking. After fixation, EGFR was visualized by anti-EGFR antibodies followed by Alexa Fluor488-conjugated antibodies and the nucleus was detected by staining with DAPI. Note the enrichment of EGFR at the plasma membrane in αPIX^WT^ overexpressing cells upon 60 min EGF stimulation (arrowheads, lower panel). 25 cells each [stably expressing CAT (control) and αPIX^WT^ cells] derived from three independent experiments have been analyzed, representative cells are shown. Scale bars, 20 μm. C. Serum-starved COS-7 cells transiently expressing HA-αPIX^WT^ were stimulated with 25 ng/ml Alexa Fluor 488-conjugated EGF (EGF488) for 15 or 60 min. Subsequently, extracellular receptor-bound EGF was removed and cells were fixed. HA-tagged αPIX was visualized by staining with anti-HA antibodies followed by Alexa Flour 546-conjugated secondary antibodies and the nucleus was detected by staining with DAPI. Dotted lines indicate αPIX-expressing cells, dashed lines indicate untransfected cells. 50 cells each (non-transfected cells and HA-αPIX^WT^ overexpressing cells) derived from three independent specimen have been examined, representative cells are shown. Scale bars, 20 μm.

We show here that αPIX is involved in the regulation of two different EGFR sorting pathways, namely the degradative and the recycling pathways. We next analyzed which αPIX function predominates under physiological conditions of the continuous presence of EGF. Cells were stimulated with EGF and the amount of intracellular EGFR was determined as a function of time. Levels of internalized EGFR were similar in αPIX^WT^ expressing and control cells after 15 and 30 min of EGF stimulation, however, after 60 min we detected strongly reduced amounts of intracellular EGFR in αPIX^WT^ cells (Fig 6A and 6B). Moreover, immunofluorescence staining of αPIX^WT^ cells demonstrated that EGFR is enriched at the plasma membrane upon 60 min EGF stimulation (Fig 6B, lower panel, arrowheads) in contrast to control cells which showed a pronounced accumulation of EGFR near the cell center (Fig 6B, lower panel). To confirm these observations by further microscopic analysis, we stimulated COS-7 cells transiently expressing αPIX^WT^ with fluorescently labeled EGF for 15 and 60 min. After 15 min, the amount of intracellular EGF was similar in αPIX^WT^ expressing and untransfected cells (Fig 6C). In contrast, αPIX^WT^ expressing cells showed strongly decreased amounts of intracellular EGF compared with untransfected COS-7 cells after 60 min EGF treatment (Fig 6C). Together, these data indicate that (i) up to 30 min of EGF treatment αPIX does not influence receptor internalization and (ii) promoting EGFR recycling and not reducing degradation is the main function of αPIX (otherwise the intracellular EGFR levels should have been increased).

**Fig 7:**
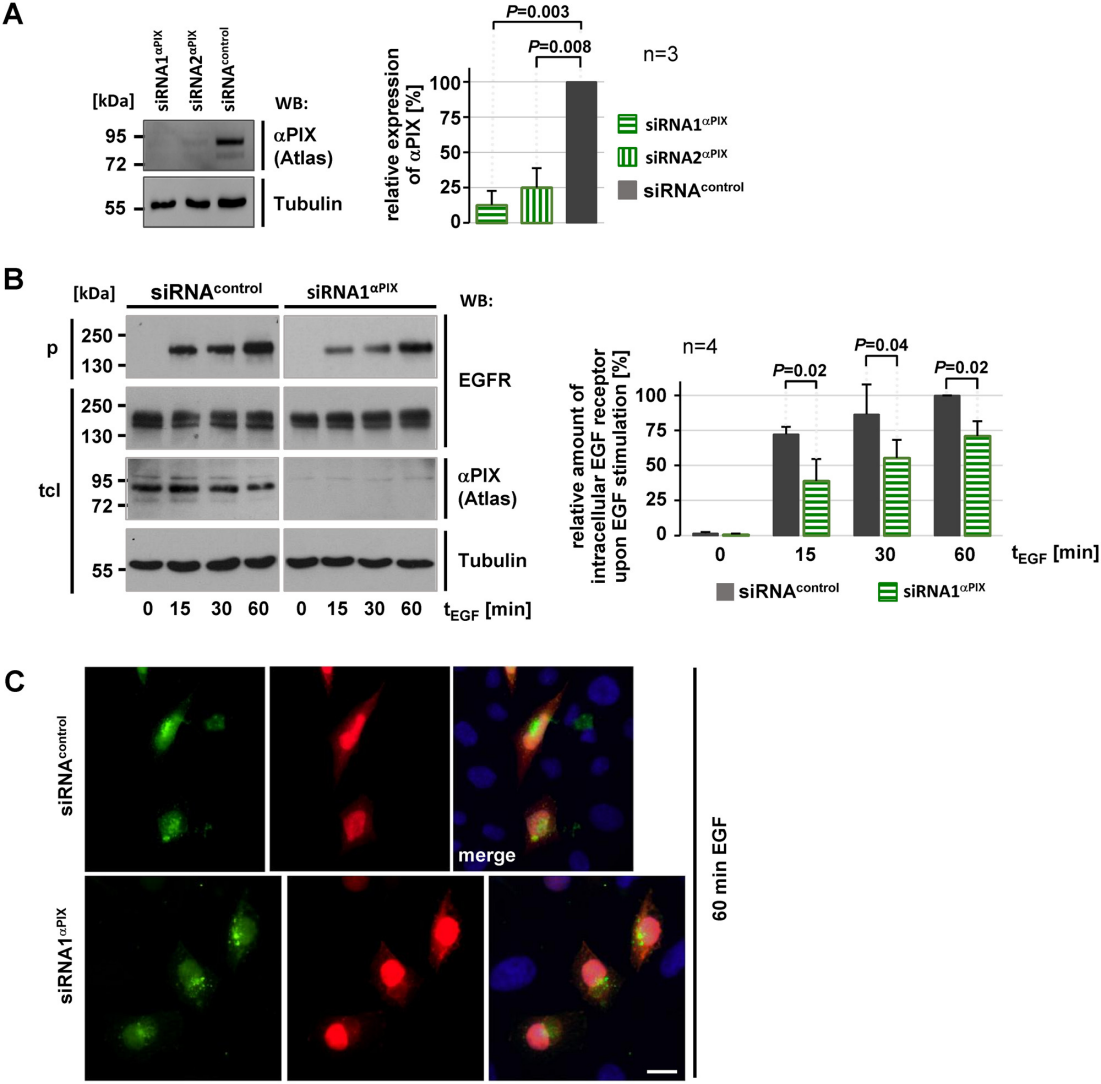
Downregulation of αPIX results in gradually increasing of intracellular EGFR levels in a steady state situation. A. CHO-K1 cells were transfected with *ARHGEF6 (αPIX)*-specific siRNAs for 48 h. GFP duplex I siRNA was used as a control. Depletion of αPIX was displayed by immunoblotting with an anti-αPIX antibody (Atlas). Re-probing of the blot with an anti-tubulin served as loading control. Representative blots from one out of three independent experiments are shown. αPIX levels in cells treated with control siRNA were considered as 100%. The data in the graph represent the mean of three (n = 3) independent experiments ± sd. *P* values were calculated by paired Student’s t-test. After 48h incubation, αPIX was depleted by 87% for (siRNA1^αPIX^) and 75% (for siRNA2^αPIX^). B. CHO-K1 cells were transfected with EGFR expression constructs together with siRNA^control^ or siRNA1^αPIX^. After 48h of incubation EGFR steady-state trafficking assays were performed as described in Fig 6A. Representative autoradiographs show EGFR levels in total cell lysates (tcl) and precipitates (p) upon SDS-PAGE and immunoblotting. αPIX depletion was verified by re-probing the membrane with anti-αPIX antibody (Atlas). Equal loading is demonstrated by probing lysates with anti-tubulin antibody. The amounts of intracellular (precipitated) EGFR were normalized to total EGFR levels and considered as 100% in cells treated with control siRNA upon 60 min EGF stimulation. Data in the graph represent the mean of four (n = 4) independent experiments ± sd. The *P* values were calculated by paired Student’s t-test. C. Immunocytochemical analysis of EGFR distribution upon knockdown of αPIX. CHO cells were co-transfected with control siRNA (siRNA^control^) or siRNA specific for αPIX (siRNA1^αPIX^), EGFR constructs, and RFP expression vectors as a transfection control. After serum starvation overnight, cells were stimulated with 25 ng/ml EGF for 60 min at 37°C to induce EGF receptor trafficking and were fixated. EGFR was visualized by anti-EGFR antibodies followed by Alexa Fluor488-conjugated antibodies and the nucleus was stained with DAPI. 25 cells each (siRNA^control^ and siRNA1^αPIX^) derived from two independent experiments have been examined, representative cells are shown. Scale bars, 10 μm.

Next we analyzed the effects of αPIX depletion on EGFR trafficking in CHO cells that endogenously express αPIX (Fig 7). We transiently reduced αPIX expression by transfection with αPIX—specific siRNAs (Fig 7A) and monitored levels of internalized EGFR upon EGF stimulation in a steady state situation (without removing EGF). Cells transfected with control siRNAs showed a strong and gradual increase of intracellular EGFR levels (Fig 7B). Intracellular EGFR also gradually increased in αPIX-depleted cells, however this increase was weaker than in controls (Fig 7B). Interestingly, immunofluorescence analyses of αPIX depleted cells demonstrated that upon 60 min of EGF stimulation intracellular EGFR also accumulated near the cell center (Fig 7C) and was not enriched at the plasma membrane as seen in αPIX overexpressing cells (see Figs 6B and 7C). In other words, levels of intracellular EGFR were generally lower in cells transfected with αPIX siRNA than in control cells (Fig 7B) but this was not a result of enhanced membrane localization of EGFR. Because we postulated that promoting EGFR recycling is the major function of αPIX, we performed pulse-chase EGFR recycling assays. Interestingly, αPIX knockdown did not affect EGFR recycling in these experiments (S4 Fig). Nonetheless, our data indicate that knockdown and overexpression of αPIX result in different intracellular EGFR levels over time (compare Figs 6A and 7B). Thus, we conclude that αPIX is necessary for maintaining intracellular EGFR levels.

### αPIX expression only slightly affects cell proliferation

EGFR induced signaling triggers a variety of cellular responses, such as proliferation, differentiation, migration and survival [56]. To monitor a possible biological consequence of altered EGFR recycling we measured proliferation in CHO cell lines stably overexpressing CAT (control), αPIX^WT^ or αPIX^W197K^ by using BrdU (Bromodeoxyuridine) incorporation assays. We observed a slightly increased BrdU incorporation in cells expressing αPIX^WT^ or αPIX^W197K^ compared to cells expressing CAT (S5 Fig). Albeit the differences did not reach statistical significance, these data may indicate that αPIX weakly stimulates proliferation. This stimulation does not depend on αPIX::c-Cbl interaction, because proliferation was even stronger in cells expressing αPIX^W197K^.

## Discussion

Trafficking of endocytozed EGFR is essential for maintaining homeostasis of EGFR-depending signaling processes [57]. If a cell needs to become desensitized for EGFR ligands, internalized receptors can be directed to the lysosome for degradation; alternatively, if signaling should be sustained or a cell needs to be resensitized for EGFR ligands, endocytozed EGFRs are recycled back to the plasma membrane [58]. Numerous proteins that modulate EGFR degradation have been identified in recent years, and many of these including βPIX, Sprouty 2, AIP1, GAPex5 and others exert their action by targeting Cbl activity [38]. On the other hand, the functioning of upstream modulators of EGFR recycling is less well studied; however several upstream modulators, such as the ARF6 and diverse RAB GTPases, effectors and regulators of these as well as various adaptor and sorting proteins have been described [59, 60]. Here we provide various lines of evidence, that αPIX controls both degradation and recycling of EGFR, thereby representing a novel bidirectional trafficking regulator. First, αPIX associates with c-Cbl, an important mediator of EGFR downregulaton. Second, αPIX::c-Cbl complex formation and αPIX/c-Cbl protein turnover depend on EGF stimulation. Third, αPIX limits EGFR ubiquitination and degradation, which depends on c-Cbl binding. Fourth, αPIX strongly increases recycling of internalized EGFR, both under experimental and physiological culture conditions, and this function depends on the GIT- and not the c-Cbl-binding capacity of αPIX. And finally fifth, here we demonstrate that promoting EGFR recycling is the major function of αPIX, and this capacity of αPIX may positively affect cell proliferation.

### Cooperation of PIX and Cbl proteins in EGFR trafficking

By mutational manipulation of amino acids essential for binding, we and recently also others [21] have shown that αPIX and c-Cbl interact via the SH3-domain and the PKPFPR-motif, respectively; moreover, here we demonstrate that this interaction is modulated by EGF stimulation. It is well established that Cbl directly and specifically determines the rate of EGFR degradation by triggering EGF-induced EGFR downregulation, which results in attenuation of EGFR-promoted signaling [38]. Accordingly, recent examples illustrated the intimate interdependency between endocytic traffic and receptor signaling events [61, 62]. βPIX, the close homologue of αPIX, negatively affects Cbl-mediated downregulation of EGFR: the interaction of the Rho GTPase Cdc42 with c-Cbl, which is mediated by βPIX, prevents c-Cbl from binding to EGFR, thereby blocking c-Cbl-catalyzed EGFR ubiquitination and downregulation [40, 41]. In line with this argumentation, our results suggest that αPIX, too, sequesters c-Cbl from EGFR which results in reduced ubiquitination and, subsequently, lysosomal sorting of EGFR. Sequestration of Cbl by βPIX and Cdc42 as well as by αPIX should be reversible; this is important to ensure normal homeostasis of EGFR trafficking and to avoid sustained activation of downstream signaling cascades. Indeed, it was previously suggested that Cbl catalyzes ubiquitination and subsequent downregulation of βPIX in response to EGF stimulation, which results in the release of Cbl from βPIX [42]. We found that αPIX degradation is induced by EGF stimulation and depends on c-Cbl binding and E3 ligase activity. Thus, we speculate that c-Cbl-mediated αPIX ubiquitination and EGF stimulation may constitute prerequisite and trigger, respectively, for αPIX degradation.

For Cbl and βPIX another regulatory mechanism has been suggested: upon EGF-dependent βPIX phosphorylation Cbl, βPIX and EGFR form a complex at the plasma membrane, which prevents Cbl from engaging essential endocytic proteins, such as CIN85 [63]; accordingly, expression of wild-type βPIX resulted in reduced EGFR internalization [41, 42]. In contrast, our data indicate that αPIX does not alter receptor internalization; in fact, independently of c-Cbl, αPIX strongly stimulated recycling of EGFR. Taken together, even though αPIX and βPIX seem to have different functions during EGFR trafficking, available data highlight their prominent roles during the regulation of endocytic pathways. It is obvious that both αPIX- and βPIX-mediated regulatory scenarios maintain EGFR signaling homeostasis: as an inhibitory molecule for the executing Cbl proteins βPIX has been attributed a rather passive function during EGFR degradation [40–42], whereas by stimulating EGFR recycling αPIX takes a very active role which is independent of c-Cbl binding (this study).

In a remarkable previous study, the role of EGFR ubiquitination as a director of EGFR recycling versus degradation was highlighted [64]. The non-ubiquitinated EGFR mutant 15KR-EGFR was not efficiently targeted to intraluminal vesicles within multivesicular bodies [64], which normally is a prerequisite for lysosomal degradation [65]. However, 15KR-EGFR showed increased recycling to the plasma membrane, which resulted from a relatively increased pool of intracellular EGFRs capable of recycling rather than from defective recycling mechanisms/pathways [64]. Following these data, αPIX might influence the number of un-ubiquitylated recyclable EGFRs by sequestration of c-Cbl; in addition and independently from c-Cbl and ubiquitylation, αPIX might stimulate recycling mechanisms/pathways.

### αPIX and EGFR recycling

An interesting aspect is how αPIX does stimulate the recycling machinery. αPIX displays GEF activity for Cdc42 and Rac1. These two GTPases are excellent *a priori* candidates for translating αPIX function to the vesicular recycling pathway because there is plenty of experimental evidence that both Cdc42 and Rac1 control vesicular trafficking by triggering spatial reorganization of the actin cytoskeleton [66, 67]. However, both expression of wild-type and GEF-deficient αPIX strongly increased recycling of EGFR to the cell surface; therefore, αPIX function during EGFR recycling is independent of its Cdc42/Rac1-specific exchange factor activity. On the other hand our experiments showed that deletion of the GIT binding domain (GBD) reversed the stimulating effect of αPIX on EGFR recycling. This observation prompted us to speculate about the underlying molecular machinery that enables αPIX to exert its function during recycling of EGFR. Via GBD αPIX and βPIX strongly interact with GIT proteins [43, 44, 68]. These multi-domain proteins function in scaffolding of signaling cascades as well as in modulation of cytoskeletal structure and membrane trafficking including endocytic EGFR transport [69]. Notably, GIT proteins have an N-terminal ARF GTPase activating (ARF-GAP) domain and affect endosomal recycling by acting on the recycling regulator ARF6 [70–73]. PIX::GIT complexes have been associated with various aspects of cell shape regulation [74]. Most interestingly, a role for a PIX::GIT-containing multi-protein complex has been described during recycling of focal adhesion components in migrating cells [75–77]. In their models the authors proposed that PIX and GIT recruit both adhesive site components and vesicles positive for the endosomal recycling markers Rab11 and sorting nexin 27. Depending on ARF6 function, these putative recycling endosomes translocate to the plasma membrane, where the PIX::GIT-containing protein complex is released [75–77]. Taken together, these data and our results indicate that αPIX may regulate endocytic recycling, i.e. trafficking between the endosomal compartment and the plasma membrane, in close cooperation with GIT family proteins. Thus, αPIX may constitute a universal factor that links vesicles with any material to be recycled (e.g. EGFR or focal adhesion components) with the GIT-ARF6 recycling machinery.

One would expect that knockdown and overexpressing of αPIX have opposite effects, however, in our study αPIX depletion by siRNAs had no effect on recycling of EGFR. This is surprising but not unusual: Previously it has been nicely reviewed that knockdown-induced functional insufficiency and overexpression-induced gain of function do not necessarily have opposite effects on cell physiology [78]. This can be explained by functional redundancy of two proteins in case of downregulation of one of these [78]. Accordingly, we can only speculate that αPIX and βPIX may be redundant in case of diminished expression of one of these; though, excess of αPIX (or βPIX) does induce a detectable phenotype.

### The working model

**Fig 8:**
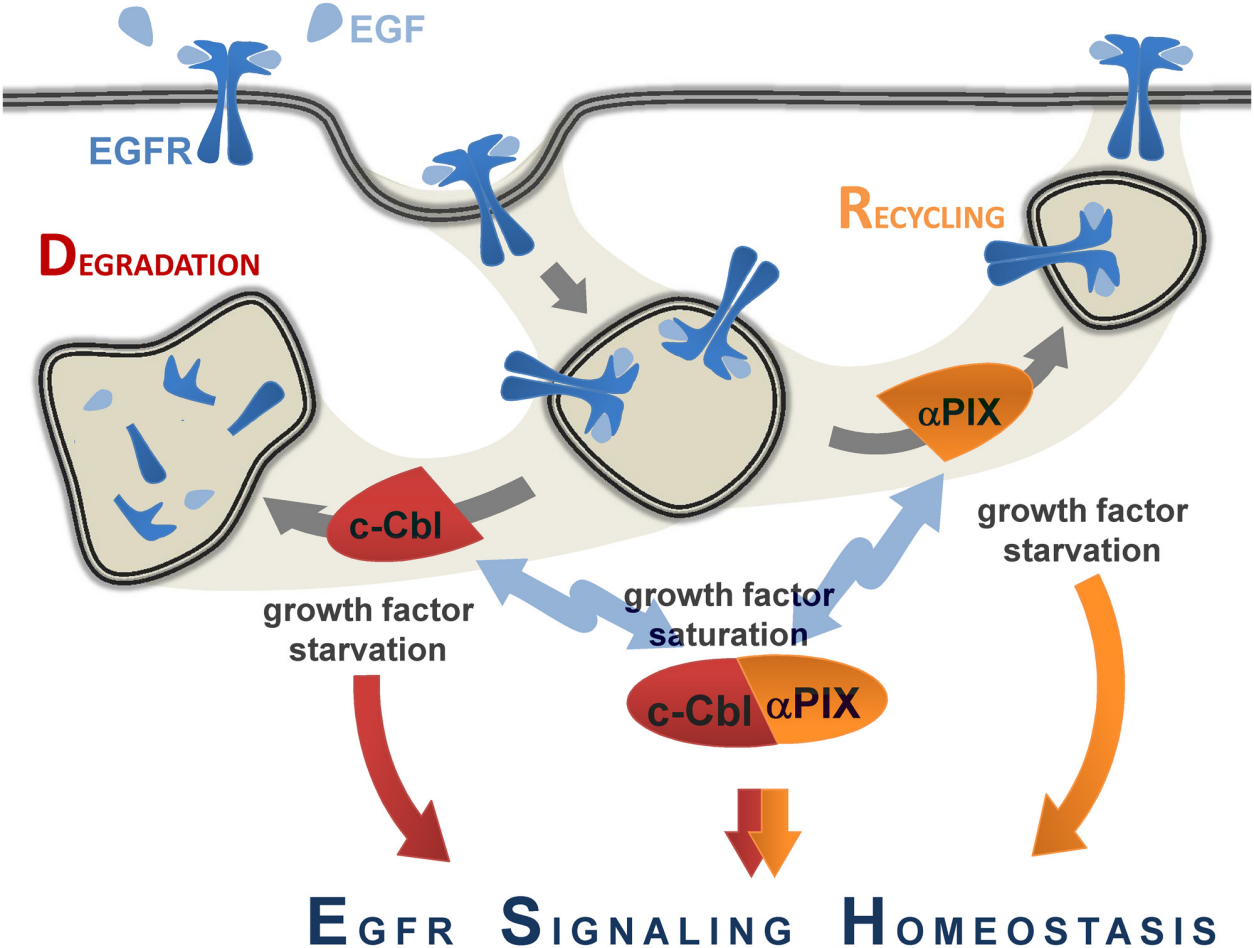
Model depicting the balancing effect of αPIX on EGFR trafficking to maintain EGFR signaling homeostasis. (For details see discussion.)

Integrating all our results, we propose that αPIX and c-Cbl are two essential components of a molecular module that controls the vesicular transport rates of specific endocytic routes, and thus, the magnitude and/or duration of the signaling response. Fig 8 shows a working model for this regulation. Uncomplexed c-Cbl promotes EGFR degradation, thereby mediating an attenuation of EGFR signaling. In contrast, uncomplexed αPIX stimulates recycling and enables a positive feedback for EGFR signaling. On the other hand, interaction of αPIX and c-Cbl results in mutual inhibition. This regulatory circuit enables a cell to compensate for harmful fluctuations in EGFR signaling and to achieve the physiologically optimal situation: (i) Under growth factor saturated conditions (i.e. +10% FBS in vitro), αPIX/c-Cbl-mediated endocytic regulation is not necessary, which is reflected by an increased αPIX::c-Cbl complex formation (Fig 8; see also Fig 2A). In line with this, at steady state, i.e. under EGF saturation, 70–80% of the EGF-occupied receptor is endosomal and only a minor receptor fraction localizes in the cell membrane [79]. (ii) Growth factor-starvation, however, results in the decay of αPIX::c-Cbl complexes (see also Fig 2A). In the absence of growth factors, cells are avid for growth factors and most of the respective receptors such as EGFR localize at the cell surface [50, 80] (see also Fig 5C and 5D). In this case unbound αPIX might promote the transport of EGFR to the surface (Fig 8). (iii) Upon EGF stimulation or any other perturbation of EGFR signaling homeostasis the cell needs to adjust EGFR signaling by adaptive response. To this end, uncomplexed c-Cbl and αPIX promote EGFR degradation and recycling, respectively, until a stable, constant condition, i.e. EGFR signaling homeostasis is preserved (Fig 8). This approximation to a homeostatic condition is associated with a gradually increase of αPIX::c-Cbl complexes (see also Fig 2A). According to this model we propose, that in or near-to perfect EGFR homeostasis c-Cbl binds and probably ubiquitinates αPIX; only perturbation of EGFR homeostasis such as induced by EGF stimulation releases αPIX from these complexes enabling αPIX-mediated receptor recycling and subsequently αPIX degradation. Notably, this is different to the model for βPIX suggested by Feng and colleagues [41] in which EGF-induced βPIX phosphorylation precedes βPIX::Cbl binding and, subsequently, βPIX ubiquitination [42]. However, in both scenarios EGF stimulation triggers the regulatory potential of the PIX proteins on endocytic traffic of EGFR. For βPIX it has been demonstrated that an EGFR-coupled protein kinase signaling cascade involving Src tyrosine-protein kinase and focal adhesion kinase (FAK) mediates its phosphorylation [41]; thus, it will be interesting whether αPIX is also phosphorylated by Src and/or FAK, too, albeit αPIX does not share the βPIX protein motif containing the phosphorylation site.

### Synopsis

In conclusion here we describe for the first time the novel function of αPIX as a potent promoter of EGFR recycling and, thus, significantly expand the knowledge about the functional implication of PIX proteins in the regulation of endocytic traffic. Our data and evidence from the literature underscore both αPIX and c-Cbl as key regulators for endocytic trafficking. Due to this prominent position in addition to their high functional potential αPIX and c-Cbl need strict regulation. We propose that this is facilitated, at least in part, by cell context-dependent association and dissociation of αPIX::c-Cbl complexes, which results in mutual inhibition and solitary activity, respectively. Certainly, the identification and examination of adequate sensor molecules that transduce changing cellular contexts, such as EGF fluctuation, to the executing αPIX::c-Cbl regulatory circuit is pending.

Our findings raise some interesting implications regarding the pathogenesis of cancer. It is well established, that malignant transformation is associated with unleashed EGFR signaling, for example due to excessive amounts of cellular EGFR [81, 82]. In addition, aberrant endocytic sorting can also lead to increased and uncontrolled receptor signaling, thereby, promoting malignant transformation [83, 84]. Thus, by shifting endocytic EGFR sorting towards recycling αPIX may enhance EGFR signaling and affect downstream cellular responses such as cell proliferation, which could explain previously reported pro-oncogenic effects of αPIX [14, 85].

## Materials and Methods

### Plasmid construction

#### Generation of N-terminal HA-tagged αPIX constructs

We amplified the coding region of human wild-type αPIX (NM_004840.2) by using αPIX-specific PCR primers and αPIX cDNA as template. Purified PCR products were cloned as *Not*I-*Eco*RI fragments in eukaryotic expression vector pMT2SM-HA. αPIX^ΔCH^, αPIX^ΔSH3^, αPIX^ΔGBD^ and αPIX^ΔCC^ constructs were previously described [16, 17]. GEF-deficient αPIX p.(L386_L387delinsRS) and c-Cbl-binding deficient αPIX p.W197K were generated by PCR-mediated mutagenesis. Two overlapping cDNA fragments were amplified with the desired mutation, applied to megaprime PCR, and PCR products were purified and cloned in pMT2SM-HA. Mutations of residues 386 and 387 in the DH (Dbl homology) domain of αPIX abolish GEF activity resulting in reduced Cdc42 and Rac1 activation as well as decreased PAK1 and JNK1 kinase activity [86, 87]. Moreover, a negative effect of αPIX p.[L386R; L387S] on the formation of lamellipodia and filopodia has been demonstrated [16].

#### Generation of N-terminal FLAG-tagged αPIX constructs and αPIX constructs for stable transfection

Wild-type and mutated pMT2SM-HA-αPIX constructs were used as templates for PCR-mediated generation of cDNA inserts which were cloned into cloning vector pENTR/D-TOPO (Life Technologies, Darmstadt, Germany) according to the protocol provided. Subsequently, these constructs were used for transferring coding regions into plasmids pFLAG-CMV4-cassetteA [17] and pEF5/FRT/V5-DEST (C-terminal V5 epitope; Life Technologies, Darmstadt, Germany) via recombination following the manufacturer’s instructions.

#### Generation of mutant c-Cbl constructs

Wild-type pRK5-c-Cbl (human; NM_005188.3) construct was kindly provided by Dr. Mirko Schmidt (Goethe University School of Medicine, Frankfurt/Main, Germany). We used this construct as a template and c-Cbl-specific PCR primers to generate wild-type c-Cbl cDNA amplicon by PCR. c-Cbl^R829A^ and c-Cbl^C381A^ were established by PCR-mediated mutagenesis. Purified PCR amplicons (c-Cbl^WT^, c-Cbl^R829A^, c-Cbl^C381A^) were cloned into pENTR/D-TOPO (Life Technologies, Darmstadt, Germany). Constructs were sequenced for integrity and used for the transfer into GATEWAY-compatible destination vector pcDNA3-DEST.

Wild-type pcDNA3-EGFR construct (human; NM_005228.3) was a kind gift of Dr. Sarah J. Parsons (University of Virginia, Virginia, USA). Flag-tagged wild-type rat Git1 (NM_031814.1) and human GIT2 (NM_057169.3) in plasmid pBK-CMV-ΔlacZ were kind gifts of Richard T. Premont (Duke University Medical Center, Durham, North Carolina, USA). Expression vector pmRFP-N1 was a kind gift of Dr. Hans-Jürgen Kreienkamp (Institute of Human Genetics, University Medical Center Hamburg-Eppendorf, Hamburg, Germany).

### Cell culture and transfection

COS-7 (african green monkey; Cat. No. ACC-60; Deutsche Sammlung von Mikroorganismen und Zellkulturen, Braunschweig, Germany) cells were cultured in 100 mm culture dishes in Dulbecco modified Eagle medium (DMEM; Life Technologies, Darmstadt, Germany). CHO-K1 were cultured in Nutrient Mixture F12 (Life Technologies, Darmstadt, Germany). Untransfected Flp-In-CHO cells (derived as a subclone from the parental Chinese hamster ovary cell line; Life Technologies, Darmstadt, Germany) were cultivated in Nutrient Mixture F12 (Life Technologies, Darmstadt, Germany) supplemented with 100 μg/ml zeocin (Life Technologies, Darmstadt, Germany). All media were supplemented with 10% fetal bovine serum (FBS; PAA—The Cell Culture Company, Cölbe, Germany) and penicillin-streptomycin (100 U/ml and 100 mg/ml, respectively; Life Technologies, Darmstadt, Germany).

COS-7 and CHO-K1 cells were transfected using Lipofectamine 2000 Reagent (Life Technologies, Darmstadt, Germany) and TurboFect (Fermentas/Thermo Scientific, St. Leon-Rot, Germany). For the generation of stable cell lines we used the Flp-In system (Life Technologies, Darmstadt, Germany). Flp-In-CHO cells were co-transfected with 1 μg of pEF5/FRT/V5-DEST containing wild-type αPIX, αPIX^W197K^, αPIX^GEF-^, or αPIX^ΔGBD^ cDNA together with 9 μg of pOG44 (Life Technologies, Darmstadt, Germany) by using Lipofectamine. Cells transfected with pEF5/FRT/V5-DEST containing chloramphenicol acetyl transferase (CAT) cDNA were used as control. Transfected cells were selected in F12 medium containing 200 μg/ml hygromycin B for approximately three weeks, and subsequently maintained in complete F12 medium supplemented with 200 μg/ml hygromycin B. Transfection of these stable Flp-In-CHO cell lines was done with TurboFect (Fermentas/Thermo Scientific, St. Leon-Rot, Germany).

### Antibodies and reagents

Following antibodies and dilutions were used: polyclonal rabbit anti-ARHGEF6/αPIX (Cat. No. HPA003578; WB 1:400) from Atlas Antibodies, Stockholm, Sweden; and anti-EEA1 (early endosome antigen 1; Cat. No. 610456; IF 1:500) from BD Biosciences, Heidelberg, Germany; rabbit polyclonal anti-Cbl (C-15; Cat. No. sc-170; WB 1:1000; IF 1:200), rabbit polyclonal anti-EGFR (clone 1005; Cat. No. sc-03; WB 1:300, IF 1:200) and mouse monoclonal anti-EGFR (clone R-1; Cat. No. sc-101; IF 1:200) from Santa Cruz, Heidelberg, Germany; mouse monoclonal anti-GAPDH (Cat. No. ab8245; WB 1:10000) from Abcam, Cambridge, UK; mouse monoclonal anti-α-Tubulin (clone DM 1A; Cat. No. T9026; WB 1:7500); rabbit polyclonal anti-HA (Cat. No. H6908; IF 1:200) and mouse monoclonal anti-FLAG M2 Peroxidase Conjugate (Cat. No. A8592; WB 1:3000) from Sigma-Aldrich, Taufkirchen, Germany; rat monoclonal anti-HA-HRP (Cat. No. 2013819; WB 1:4000) from Roche, Mannheim, Germany; mouse monoclonal anti-V5-HRP (Cat. No. R96125; WB 1:5000) from Life Technologies, Darmstadt, Germany; epidermal growth factor (EGF) complexed with Alexa Fluor 488 was purchased from Molecular Probes/Life Technologies, Darmstadt, Germany and unlabelled human epidermal growth factor from Sigma-Aldrich, Taufkirchen, Germany.

### Co-immunoprecipitations

#### Co-immunoprecipitation of ectopically expressed proteins

If needed, transiently transfected COS-7 cells were serum-starved and treated with EGF before cell lysis. Cells were lysed in ice-cold cell lysis buffer (150 mM Tris-HCl, pH 8.0; 50 mM NaCl; 1 mM EDTA; 0.5% Nonidet P40; complete Mini Protease Inhibitors [Roche, Mannheim, Germany]; 0.7 mg/ml Pepstatin), and cell lysates were clarified by centrifugation. The supernatants were transferred to 40 μl EZview Red Anti-HA Affinity Gel (Roche, Mannheim, Germany) and incubated for 2 h at 4°C on a rotator. Precipitates were collected by repeated centrifugation and washing with TBS (50 mM Tris-HCl, pH 7.4; 150 mM NaCl), resuspended in sample buffer (33% glycerol; 80 mM Tris-HCl, pH 6.8; 0.3 M Dithiothreitol; 6.7% sodium dodecyl sulphate; 0.1% bromophenol blue) and subjected to SDS-PAGE and immunoblotting.

#### Co-immunoprecipitation of endogenous proteins from CHO-K1 cells

CHO-K1 cells were harvested with ice-cold IP lysis buffer and cell debris was removed by centrifugation. Next, supernatants were pre-cleared by incubation with protein A-conjugated agarose (Roche, Mannheim, Germany) and centrifugation, 5 μg specific antibodies were added to the pre-cleared lysates and solutions were incubated for 3 h at 4°C on a rotator. As controls non-specific normal IgG antibodies raised in the same host as the respective specific antibodies were added to pre-cleared lysates. Subsequently, fresh protein A-conjugated agarose beads were added and the solution was incubated overnight at 4°C on a rotator. Beads-antibody-protein complexes were collected by centrifugation, washed five times with IP lysis buffer, and cell lysates as well as precipitates were subjected to SDS-PAGE and western blot analysis.

### EGFR ubiquitination assay

COS-7 cells were transiently transfected with c-Cbl expression constructs together with FLAG-tagged αPIX, HA-tagged ubiquitin and EGFR expression constructs. Transfected cells were incubated in Opti-MEM (Life Technologies, Darmstadt, Germany) overnight. Next day, cells were stimulated with 20 ng/ml EGF in starvation medium (DMEM supplemented with 0.1% serum) for 30 min at 37°C. Cells were washed with ice-cold 1×PBS and harvested in ice-cold RIPA buffer containing protease inhibitor cocktail and 10 mM N-ethylmaleimide (Sigma-Aldrich, Taufkirchen, Germany) to inhibit deubiquitinating enzymes. Next, the cell debris was removed by centrifugation and aliquots of the total cell lysates were kept for immunoblotting. The remaining supernatants were incubated with 1 μg of anti-EGFR (Santa Cruz Biotechnology) for 2 hr at 4°C, followed by incubation with protein A-agarose beads (Roche) overnight at 4°C. EGFR coupled to agarose beads were collected by centrifugation for 30 sec at 12000 g at 4°C, and agarose pellets were washed three times with ice-cold Triton lysis buffer (1% Triton X-100 [v/v], 150 mM NaCl, 5 mM EDTA, 50 mM HEPES, pH 7.5) supplemented with 0.05% (w/v) SDS. Precipitates and total cell lysates were separated on SDS-polyacrylamide gels, transferred to PVDF membranes, and subjected to immunodetection.

### EGFR trafficking assays

#### EGFR pulse-chase trafficking assay

(Fig 3). Stable Flp-In-CHO cell lines were transiently transfected with EGFR constructs and incubated under serum starved (0.1% FBS) culture conditions overnight. Next day, cells were cooled on ice, washed three times with ice-cold HBSS, and cell surface proteins were biotinylated using 0.5 mg/ml biotin (EZ-Link Sulfo-NHS-SS-Biotin, Thermo Scientific, St. Leon-Rot, Germany) in HBSS for 15 min at 4°C. Subsequently, biotin was quenched by three successive washes in HBSS with 5 mM Tris-HCl (pH 7.4) and cells were rinsed with ice-cold PBS. Internalization of biotinylated EGF receptors was induced by incubation in medium supplemented with 25 ng/ml EGF for 30 min at 37°C. Cells were transferred on ice and residual surface proteins were de-biotinylated by incubation in ice-cold stripping buffer (50 mM glutathione, 75 mM NaCl, 1 mM EDTA, 10% FBS, and 75 mM NaOH). To induce and record trafficking of intracellular EGFR, cells were rewarmed to 37°C in pre-warmed HBSS (“chase medium”) for various times. Subsequently, cells were transferred on ice and incubated a second time in cold glutathione stripping buffer to de-biotinylate recycled cell-surface proteins. Cells were rinsed with ice-cold PBS and lysed with ice-cold RIPA buffer (150 mM NaCl, 1% NP-40, 0.5% sodium deoxycholate, 0.1% SDS, 50 mM Tris, pH 8). As controls (corresponding to 0 min in Fig 3) parallel cultures were lysed in RIPA buffer before rewarming/de-biotinylation. After sedimentation of cell debris, 50 μl streptavidin-conjugated agarose beads (Sigma-Aldrich, Taufkirchen, Germany) each was added to the supernatants and solutions were incubated ≥ 2 hours at 4°C on a rotator. Intracellular, biotin-labelled EGFR fractions were precipitated from cell extracts by centrifugation and precipitates were washed twice with RIPA buffer. Finally, both raw lysates and precipitates were subjected to SDS-PAGE and western blot analysis.

#### EGFR pulse-chase degradation assay

(Fig 4). The experimental procedure was essentially the same as described above (“EGFR pulse-chase trafficking assay”) with following exceptions: (i) To block EGFR recycling, pulse and chase media were supplemented with 0.3 mM of the recycling inhibitor primaquine (Sigma-Aldrich, Taufkirchen, Germany). (ii) As controls (corresponding to 0 min in Fig 4) parallel cultures were lysed in RIPA buffer before rewarming/de-biotinylation.

#### EGFR pulse-chase recycling assay

(Fig 5A and 5B; S3, S4 Figs). The experimental procedure was essentially the same as described above (“EGFR pulse-chase trafficking assay”) with following exceptions: (i) 24 hours after transfection cells lines were pre-incubated in medium supplemented with lysosomal degradation inhibitors leupeptin (100 μM) and pepstatin A (100 μM) for additional 24 h. (ii) Subsequent EGF stimulation and de-biotinylation of residual surface proteins (see above), parallel cultures were subjected to 1, 2 or 3 cycles of 2 min rewarming to 37°C and de-biotinylation (as described above) of recycled receptors. (iii) As controls (corresponding to 0 cycles in Fig 5A and 5B, S3 and S4 Figs) parallel cultures were lysed in RIPA buffer before rewarming/de-biotinylation.

#### EGFR steady-state cell surface assay

(Fig 5C and 5D). Stable Flp-In-CHO cell lines were transiently transfected with EGFR expression constructs and incubated under serum starved (0.1% FBS) culture conditions overnight. Subsequently, cells were stimulated with starvation medium supplemented with 25 ng/ml EGF for various times. Cells were transferred to ice, rinsed three times with ice-cold HBSS and cell surface proteins were biotinylated using 0.5 mg/ml biotin in HBSS for 15 min at 4°C. After excess biotin was removed by washing twice with 5 mM Tris-HCl (pH 7.4) in HBSS, cells were rinsed with ice-cold PBS and lysed in RIPA buffer. As controls (corresponding to 0 min in Fig 5C and 5D) unstimulated cultures were lysed in RIPA buffer. Next, cell debris was removed by centrifugation and streptavidin-conjugated agarose beads (50 μl each) were added to the supernatants. Solutions were incubated ≥ 2 hours at 4°C, biotin-labelled surface EGFR fractions coupled to agarose beads were collected by centrifugation and precipitates were washed twice with ice-cold RIPA buffer. Finally, total cell lysates and precipitates were subjected to SDS-PAGE and western blot analysis.

#### EGFR steady-state trafficking assay

(Figs 6A and 7B). Stable Flp-In-CHO cell lines as well as siRNA treated CHO cells were transiently transfected with EGFR expression constructs and incubated under serum starved culture conditions overnight. Cells were transferred to ice, rinsed three times with ice-cold HBSS and cell surface proteins were biotinylated using 0.5 mg/ml biotin in HBSS for 15 min at 4°C. Unbound biotin was quenched by washing three times with 5 mM Tris-HCl (pH 7.4) in HBSS. Internalization of biotinylated EGF receptors was induced by stimulation with 25 ng/ml EGF in starvation medium for various times. Cells were transferred on ice and residual surface proteins were de-biotinylated by incubation in ice-cold glutathione stripping buffer. After washing with PBS cells were lysed in RIPA buffer; as controls (corresponding to 0 min in Figs 6A and 7B) and to demonstrate the efficiency of biotin-stripping, unstimulated cultures were lysed in RIPA buffer. The further proceeding was as described above (“EGFR steady-state cell surface assay”), however, here intracellular EGFR fractions were collected in the precipitates.

### RNAi-mediated αPIX knockdown

To analyze EGFR trafficking in αPIX depleted cells, we applied RNA interference for down-regulation of native αPIX expression in CHO-K1 cells (Fig 7A). We used two different *ARHGEF6* (αPIX)-specific siRNAs to exclude off-target effects (target sequences: siRNA1^αPIX^ 5-CAACCTAAGTGGTGATAAATT-3; siRNA2^αPIX^ 5-AAAGAAAGACTGAGCGAAATT-3; GE Healthcare, Dharmacon, Lafayette, CO, USA). Cells were co-transfected on 100 mm dishes using 30 μl Lipofectamine 2000 Reagent, 6 μg EGFR expression constructs and 500 pmol αPIX-specific siRNA and cultured cells for 48 h. GFP Duplex I control siRNA (GE Healthcare Dharmacon Inc.) was used as negative control. Upon 48h of αPIX down-regulation, we performed EGFR steady-state trafficking assays (Fig 7B) as well as EGFR pulse-chase recycling assays (S4 Fig) as described above.

### Immunocytochemistry

COS-7 and CHO cells were cultivated on coverslips and, if needed, transiently transfected with expression constructs. To track EGF internalization, COS-7 cells were serum starved for 24 h and stimulated with 25 ng/ml fluorescently labelled EGF in starvation medium for 15 or 60 min followed by an acidic wash to remove non-internalized and recycled EGF from plasma membrane standing EGFR (Fig 6C). To analyse the morphology of EEA-positive vesicular structures (Fig 3C), serum-starved COS-7 cells were pulsed with 25 ng/ml EGF for 30 min at 37°C, rinsed with PBS and chased in starvation medium for 30 min. To examine the cellular distribution of EGFR, CHO cells transfected with control siRNA (siRNA^control^) or siRNA specific for αPIX (siRNA1^αPIX^) (Fig 7C) and Flp-In-CHO stably expressing CAT or αPIX^WT^ (Fig 6B) were used. Co-transfection of an RFP expression vector served as control for successful siRNA transfection of CHO cells. Both, siRNA transfected CHO cells and stably expressing Flp-In-CHO were transiently transfected with EGFR constructs, serum starved overnight and stimulated with EGF for 15 or 60 min. Subsequently, cells were rinsed with PBS, fixed with 4% paraformaldehyde (Sigma-Aldrich, Taufkirchen, Germany) in PBS and washed three times with PBS. After treatment with permeabilization/blocking solution (2% BSA, 3% goat serum, 0.5% Nonidet P40 in PBS) cells were incubated in antibody solution (3% goat serum and 0.1% Nonidet P40 in PBS) containing appropriate primary antibodies. Cells were washed with PBS and incubated with Fluorophore-conjugated secondary antibodies (Alexa Fluor Dyes; Life Technologies, Darmstadt, Germany) in antibody solution. After extensive washing with PBS cells were embedded in mounting solution (25% Mowiol 4–88 in PBS mixed with 5% Propyl gallate in PBS/Glycerol in a ratio of 4:1) on microscopic slides. Cells were examined with a Leica DMIRE2 confocal microscope equipped with an HCX PL APO 63x/1.32 oil immersion objective lens.

### BrdU (Bromodeoxyuridine) Cell Proliferation Assay

We used BrdU Cell Proliferation Assay Kit (Cat. No. #6813, Cell Signaling Technology, Danvers, MA, USA) to investigate proliferation in stable CHO cell lines. 12,500 cells were seeded in 100 μl starvation medium (F12 medium, 0.1% FBS, 100 U/ml penicillin and 100 mg/ml streptomycin) and incubated at 37°C for 24h hours to synchronize the cell cycle. Medium was then changed to 1x BrdU solution prepared in regular growth medium (F12 medium, 10% FBS, 100 U/ml penicillin and 100 mg/ml streptomycin) and incubated for 6h at 37°C to induce proliferation and incorporation of BrdU during S-Phase. Subsequent procedure was performed according to the manufacturer’s instructions. The BrdU incorporation was measured at 450 nm with the Epoch Microplate Spectrophotometer (BioTek, Bad Friedrichshall, Germany) using the Gen5 Data Analysis software (BioTek, Bad Friedrichshall, Germany).

### Statistical analysis

Signals on autoradiographs from three to six independent experiments were quantified by densitometric analysis using the ImageJ software (NIH; http://rsb.info.nih.gov/ij/index.html). Relative amounts of αPIX::c-Cbl complexes (Fig 2A) and of αPIX and Cbl (Fig 2B and 2C) were determined as described in the figure legend. Two-tailed paired (Fig 2A), one-tailed paired (Fig 2B) and one-tailed unpaired (Fig 2C) Student’s t-tests were used to determine the significance of the difference of the mean values between indicated times of EGF stimulation. Relative intracellular or surface EGFR levels were assessed as described in the figure legends (Figs 3A and 3B; 4A and 4B; 5A–5D and 6A and 7B). Two-tailed unpaired Student’s t-tests were used to determine the significance of the difference of the mean values between individual cell lines (Figs 3B, 4B, 5B and 5D), whereas a two-tailed paired Student’s t-test was used to determine the significance of the average difference between individual cell lines (Figs 6A and 7B). Relative ubiquitylation of EGFR was assessed as described in the legend to Fig 4E and a two-tailed paired Student’s t-test was used to determine the significance of the difference of the mean values between indicated cell cultures. BrdU incorporation was assessed as described in the legend to S5 Fig and a paired Student’s t-test was used to determine the significance of the difference of the mean values between individual cell lines. Quantification of αPIX depletion was assessed as described in the legend to Fig 7A. One-tailed paired Student`s t-test were used to determine the significance of αPIX knockdown by αPIX-specific siRNAs compared to control siRNAs (Fig 7A). Values were considered significant at *P* (P-values) <0.05.

## Supporting Information

S1 Fig αPIX GBD is essential for binding to GIT proteins. COS-7 cells were transiently co-transfected with the indicated expression constructs. For control purpose empty HA-vector was used. HA-tagged αPIX was immunoprecipitated from cell extracts by using anti-HA-conjugated agarose beads. After SDS-PAGE and western blotting, immunoprecipitates (IP) and total cell lysates (tcl) were probed with anti-HA and anti-FLAG antibodies. The HA-membrane was re-probed using anti-GAPDH antibodies to control for equal loading. Both, Flag-tagged rat Git1 and human GIT2 well co-precipitated with HA-αPIX^wt^ (wild-type). In contrast, deletion of αPIX GBD (αPIX^ΔGBD^) abolished co-immunoprecipitation of FLAG-Git1 and FLAG-GIT2 (top panel). All other tested αPIX deletion variants (αPIX^ΔCH^, αPIX^ΔCC^, αPIX^ΔSH3^; please see Fig 1B) did not affect binding with FLAG-Git1 or FLAG-GIT2.
(TIF)

S2 Fig Stable expression of different PIX protein variants in Flp-In-CHO cells. Cell lines stably overexpressing the indicated V5-tagged αPIX protein variants or V5-tagged CAT (control) were cultivated under basal growth conditions. Cell extracts were subjected to western blot analysis using anti-V5 antibodies. Tubulin served as a loading control.
(TIF)

S3 Fig GIT2 rescues stimulation of recycling in αPIX^ΔGBD^ cells. CHO cells stably expressing αPIX^ΔGBD^ were co-transfected with EGFR and GIT2 expression constructs followed by incubation in starvation medium supplemented with pepstatin A and leupeptin to inhibit lysosomal degradation. Surface proteins were biotinylated and cells were stimulated with 25 ng/ml EGF for 30 min at 37°C to induce EGF receptor trafficking. Subsequently, cells were transferred to 4°C and residual surface biotin was removed. Parallel cultures were subjected to 1, 2 or 3 cycles of 2 min rewarming at 37°C and de-biotinylation of recycled receptors. Intracellular biotinylated proteins were precipitated from cell extracts. Parallel cultures were harvested without rewarming/de-biotinylation (0 cycles). Total cell lysates (tcl) and precipitates (p) were subjected to SDS-PAGE and immunoblotting using anti-EGFR antibodies. Expression of FLAG-tagged GIT2 was verified by immunoblotting of tcl with anti-FLAG antibodies. Tubulin served as a loading control. We observed a reasonably constant intracellular EGFR pool over time (please see 1^st^, 2^nd^ and 3^rd^ cycle of rewarming) in cells expressing αPIX^ΔGBD^ but not GIT2 (FLAG-vector). In contrast the amount of intracellular EGFR gradually decreased in cells co-expressing FLAG-GIT2, suggesting that in the regulation of EGFR recycling GIT2 acts downstream of αPIX.
(TIF)

S4 Fig αPIX downregulation does not affect EGFR recycling. CHO-K1 cells were transfected with EGFR expression constructs and siRNA1^αPIX^, siRNA2^αPIX^ or control siRNA (siRNA^control^). 24h post transfection cells were incubated in starvation medium supplemented with pepstatin A and leupeptin for additional 24h to inhibit lysosomal degradation. Subsequently, surface proteins were biotinylated, and cells were treated with 25 ng/ml EGF for 30 min at 37°C to induce EGFR internalization. Cell surface-bound biotin was stripped off and cells were subjected to up to three cycles of rewarming to 37°C for 2 min and de-biotinylation of recycled receptors. Parallel cultures were harvested without rewarming/de-biotinylation (0 cycles). Intracellular biotinylated receptors were precipitated from cell extracts by streptavidin affinity gel. Total cell extracts (tcl) and precipitates (p) were analyzed by immunoblotting using anti-EGFR, anti-αPIX and anti-Tubulin antibodies.
(TIF)

S5 Fig αPIX is a weak promoter of cell proliferation. 12.500 CHO cells stably expressing CAT (control), αPIX^WT^ or αPIX^W197K^ were starved for 24h hours to synchronize the cell cycle. Subsequently, cells were stimulated with regular growth medium containing BrdU for 6h to induce proliferation and incorporation of BrdU during S-Phase. BrdU incorporation was measured photometrically. Graphs represent relative absorbance measured at 450 nm. For quantification the absorption of a cell-free well was subtracted and the mean value of CAT expressing control cells was used for normalization. Data represent the mean of four (n = 4) independent experiments ± sd. P values were calculated by paired Student’s t-test.
(TIF)

## References

[1] Kutsche K, Yntema H, Brandt A, Jantke I, Nothwang HG, Orth U, et al Mutations in ARHGEF6, encoding a guanine nucleotide exchange factor for Rho GTPases, in patients with X-linked mental retardation. Nat Genet. 2000;26(2):247–50. Epub 2000/10/04. 10.1038/80002 . 11017088

[2] Bagrodia S, Taylor SJ, Jordon KA, Van Aelst L, Cerione RA. A novel regulator of p21-activated kinases. J Biol Chem. 1998;273(37):23633–6. . 9726964 10.1074/jbc.273.37.23633

[3] Manser E, Loo TH, Koh CG, Zhao ZS, Chen XQ, Tan L, et al PAK kinases are directly coupled to the PIX family of nucleotide exchange factors. Mol Cell. 1998;1(2):183–92. 9659915 10.1016/s1097-2765(00)80019-2

[4] Baird D, Feng Q, Cerione RA. The Cool-2/alpha-Pix protein mediates a Cdc42-Rac signaling cascade. Curr Biol. 2005;15(1):1–10. . 15649357 10.1016/j.cub.2004.12.040

[5] Feng Q, Baird D, Cerione RA. Novel regulatory mechanisms for the Dbl family guanine nucleotide exchange factor Cool-2/alpha-Pix. The EMBO journal. 2004;23(17):3492–504. Epub 2004/08/13. 10.1038/sj.emboj.7600331 15306850 PMC516622

[6] Koh CG, Manser E, Zhao ZS, Ng CP, Lim L. Beta1PIX, the PAK-interacting exchange factor, requires localization via a coiled-coil region to promote microvillus-like structures and membrane ruffles. J Cell Sci. 2001;114(Pt 23):4239–51. . 11739656 10.1242/jcs.114.23.4239

[7] Feng Q, Albeck JG, Cerione RA, Yang W. Regulation of the Cool/Pix proteins: key binding partners of the Cdc42/Rac targets, the p21-activated kinases. J Biol Chem. 2002;277(7):5644–50. Epub 2001/12/14. 10.1074/jbc.M107704200 . 11741931

[8] Ramakers GJ, Wolfer D, Rosenberger G, Kuchenbecker K, Kreienkamp HJ, Prange-Kiel J, et al Dysregulation of Rho GTPases in the alphaPix/Arhgef6 mouse model of X-linked intellectual disability is paralleled by impaired structural and synaptic plasticity and cognitive deficits. Human molecular genetics. 2012;21(2):268–86. Epub 2011/10/13. 10.1093/hmg/ddr457 . 21989057

[9] Rossman KL, Der CJ, Sondek J. GEF means go: turning on RHO GTPases with guanine nucleotide-exchange factors. Nat Rev Mol Cell Biol. 2005;6(2):167–80. . 15688002 10.1038/nrm1587

[10] Etienne-Manneville S, Hall A. Rho GTPases in cell biology. Nature. 2002;420(6916):629–35. . 12478284 10.1038/nature01148

[11] Heasman SJ, Ridley AJ. Mammalian Rho GTPases: new insights into their functions from in vivo studies. Nat Rev Mol Cell Biol. 2008;9(9):690–701. 10.1038/nrm2476 18719708

[12] Jaffe AB, Hall A. Rho GTPases: biochemistry and biology. Annu Rev Cell Dev Biol. 2005;21:247–69. . 16212495 10.1146/annurev.cellbio.21.020604.150721

[13] Stofega MR, Sanders LC, Gardiner EM, Bokoch GM. Constitutive p21-activated kinase (PAK) activation in breast cancer cells as a result of mislocalization of PAK to focal adhesions. Mol Biol Cell. 2004;15(6):2965–77. . 15047871 10.1091/mbc.E03-08-0604 PMC420118

[14] Hua KT, Tan CT, Johansson G, Lee JM, Yang PW, Lu HY, et al N-alpha-acetyltransferase 10 protein suppresses cancer cell metastasis by binding PIX proteins and inhibiting Cdc42/Rac1 activity. Cancer Cell. 2011;19(2):218–31. Epub 2011/02/08. 10.1016/j.ccr.2010.11.010 . 21295525

[15] Yoshimi R, Yamaji S, Suzuki A, Mishima W, Okamura M, Obana T, et al The gamma-parvin-integrin-linked kinase complex is critically involved in leukocyte-substrate interaction. J Immunol. 2006;176(6):3611–24. Epub 2006/03/07. . 16517730 10.4049/jimmunol.176.6.3611

[16] Rosenberger G, Gal A, Kutsche K. AlphaPIX associates with calpain 4, the small subunit of calpain, and has a dual role in integrin-mediated cell spreading. J Biol Chem. 2005;280(8):6879–89. Epub 2004/12/22. 10.1074/jbc.M412119200 . 15611136

[17] Rosenberger G, Jantke I, Gal A, Kutsche K. Interaction of alphaPIX (ARHGEF6) with beta-parvin (PARVB) suggests an involvement of alphaPIX in integrin-mediated signaling. Human molecular genetics. 2003;12(2):155–67. Epub 2002/12/25. . 12499396 10.1093/hmg/ddg019

[18] Rosenberger G, Kutsche K. AlphaPIX and betaPIX and their role in focal adhesion formation. Eur J Cell Biol. 2006;85(3–4):265–74. Epub 2005/12/13. 10.1016/j.ejcb.2005.10.007 . 16337026

[19] Gringel A, Walz D, Rosenberger G, Minden A, Kutsche K, Kopp P, et al PAK4 and alphaPIX determine podosome size and number in macrophages through localized actin regulation. Journal of cellular physiology. 2006;209(2):568–79. Epub 2006/08/10. 10.1002/jcp.20777 . 16897755

[20] Korthals M, Schilling K, Reichardt P, Mamula D, Schluter T, Steiner M, et al alphaPIX RhoGEF supports positive selection by restraining migration and promoting arrest of thymocytes. J Immunol. 2014;192(7):3228–38. 10.4049/jimmunol.1302585 . 24591366

[21] Seong MW, Park JH, Yoo HM, Yang SW, Oh KH, Ka SH, et al c-Cbl regulates alphaPix-mediated cell migration and invasion. Biochemical and biophysical research communications. 2014;455(3–4):153–8. 10.1016/j.bbrc.2014.10.129 . 25450678

[22] Li Z, Hannigan M, Mo Z, Liu B, Lu W, Wu Y, et al Directional sensing requires G beta gamma-mediated PAK1 and PIX alpha-dependent activation of Cdc42. Cell. 2003;114(2):215–27. . 12887923 10.1016/s0092-8674(03)00559-2

[23] Mazaki Y, Hashimoto S, Tsujimura T, Morishige M, Hashimoto A, Aritake K, et al Neutrophil direction sensing and superoxide production linked by the GTPase-activating protein GIT2. Nature immunology. 2006;7(7):724–31. Epub 2006/05/23. 10.1038/ni1349 . 16715100

[24] Totaro A, Tavano S, Filosa G, Gartner A, Pennucci R, Santambrogio P, et al Biochemical and functional characterization of alphaPIX, a specific regulator of axonal and dendritic branching in hippocampal neurons. Biol Cell. 2012 Epub 2012/05/03. 10.1111/boc.201200006 . 22554054

[25] Node-Langlois R, Muller D, Boda B. Sequential implication of the mental retardation proteins ARHGEF6 and PAK3 in spine morphogenesis. J Cell Sci. 2006;119(Pt 23):4986–93. Epub 2006/11/16. 10.1242/jcs.03273 . 17105769

[26] Phee H, Abraham RT, Weiss A. Dynamic recruitment of PAK1 to the immunological synapse is mediated by PIX independently of SLP-76 and Vav1. Nature immunology. 2005;6(6):608–17. . 15864311 10.1038/ni1199

[27] Missy K, Hu B, Schilling K, Harenberg A, Sakk V, Kuchenbecker K, et al AlphaPIX Rho GTPase guanine nucleotide exchange factor regulates lymphocyte functions and antigen receptor signaling. Mol Cell Biol. 2008;28(11):3776–89. Epub 2008/04/02. 10.1128/MCB.00507-07 18378701 PMC2423308

[28] Yoshii S, Tanaka M, Otsuki Y, Wang DY, Guo RJ, Zhu Y, et al alphaPIX nucleotide exchange factor is activated by interaction with phosphatidylinositol 3-kinase. Oncogene. 1999;18(41):5680–90. Epub 1999/10/19. 10.1038/sj.onc.1202936 . 10523848

[29] Kong KF, Fu G, Zhang Y, Yokosuka T, Casas J, Canonigo-Balancio AJ, et al Protein kinase C-eta controls CTLA-4-mediated regulatory T cell function. Nature immunology. 2014;15(5):465–72. 10.1038/ni.2866 24705298 PMC4040250

[30] Llavero F, Urzelai B, Osinalde N, Galvez P, Lacerda HM, Parada LA, et al Guanine nucleotide exchange factor alphaPIX leads to activation of the Rac 1 GTPase/Glycogen phosphorylase pathway in Interleukin (IL)-2-stimulated T cells. J Biol Chem. 2015 10.1074/jbc.M114.608414 . 25694429 PMC4423703

[31] Flanders JA, Feng Q, Bagrodia S, Laux MT, Singavarapu A, Cerione RA. The Cbl proteins are binding partners for the Cool/Pix family of p21-activated kinase-binding proteins. FEBS Lett. 2003;550(1–3):119–23. Epub 2003/08/26. . 12935897 10.1016/s0014-5793(03)00853-6

[32] Schmidt MH, Dikic I. The Cbl interactome and its functions. Nat Rev Mol Cell Biol. 2005;6(12):907–19. . 16227975 10.1038/nrm1762

[33] Thien CB, Langdon WY. Cbl: many adaptations to regulate protein tyrosine kinases. Nat Rev Mol Cell Biol. 2001;2(4):294–307. . 11283727 10.1038/35067100

[34] Thien CB, Langdon WY. Negative regulation of PTK signalling by Cbl proteins. Growth factors (Chur, Switzerland). 2005;23(2):161–7. . 16019438 10.1080/08977190500153763

[35] Weissman AM. Themes and variations on ubiquitylation. Nat Rev Mol Cell Biol. 2001;2(3):169–78. . 11265246 10.1038/35056563

[36] Staub O, Rotin D. Role of ubiquitylation in cellular membrane transport. Physiological reviews. 2006;86(2):669–707. . 16601271 10.1152/physrev.00020.2005

[37] Dikic I, Szymkiewicz I, Soubeyran P. Cbl signaling networks in the regulation of cell function. Cell Mol Life Sci. 2003;60(9):1805–27. . 14523545 10.1007/s00018-003-3029-4 PMC11146075

[38] Sorkin A, Goh LK. Endocytosis and intracellular trafficking of ErbBs. Exp Cell Res. 2009;315(4):683–96. . 19278030 10.1016/j.yexcr.2008.07.029 PMC2605728

[39] Swaminathan G, Tsygankov AY. The Cbl family proteins: ring leaders in regulation of cell signaling. J Cell Physiol. 2006;209(1):21–43. . 16741904 10.1002/jcp.20694

[40] Wu WJ, Tu S, Cerione RA. Activated Cdc42 sequesters c-Cbl and prevents EGF receptor degradation. Cell. 2003;114(6):715–25. . 14505571 10.1016/s0092-8674(03)00688-3

[41] Feng Q, Baird D, Peng X, Wang J, Ly T, Guan JL, et al Cool-1 functions as an essential regulatory node for EGF receptor- and Src-mediated cell growth. Nat Cell Biol. 2006;8(9):945–56. . 16892055 10.1038/ncb1453

[42] Schmidt MH, Husnjak K, Szymkiewicz I, Haglund K, Dikic I. Cbl escapes Cdc42-mediated inhibition by downregulation of the adaptor molecule betaPix. Oncogene. 2006;25(21):3071–8. . 16407834 10.1038/sj.onc.1209329

[43] Bagrodia S, Bailey D, Lenard Z, Hart M, Guan JL, Premont RT, et al A tyrosine-phosphorylated protein that binds to an important regulatory region on the cool family of p21-activated kinase-binding proteins. J Biol Chem. 1999;274(32):22393–400. . 10428811 10.1074/jbc.274.32.22393

[44] Turner CE, Brown MC, Perrotta JA, Riedy MC, Nikolopoulos SN, McDonald AR, et al Paxillin LD4 motif binds PAK and PIX through a novel 95-kD ankyrin repeat, ARF-GAP protein: A role in cytoskeletal remodeling. J Cell Biol. 1999;145(4):851–63. . 10330411 10.1083/jcb.145.4.851 PMC2133183

[45] Schlenker O, Rittinger K. Structures of dimeric GIT1 and trimeric beta-PIX and implications for GIT-PIX complex assembly. J Mol Biol. 2009;386(2):280–9. 10.1016/j.jmb.2008.12.050 . 19136011

[46] Waterman H, Levkowitz G, Alroy I, Yarden Y. The RING finger of c-Cbl mediates desensitization of the epidermal growth factor receptor. J Biol Chem. 1999;274(32):22151–4. . 10428778 10.1074/jbc.274.32.22151

[47] Lin Q, Yang W, Cerione RA. Measurement of epidermal growth factor receptor turnover and effects of Cdc42. Methods Enzymol. 2006;406:614–25. Epub 2006/02/14. 10.1016/S0076-6879(06)06048-4 . 16472692

[48] Sorkin A, Duex JE. Quantitative analysis of endocytosis and turnover of epidermal growth factor (EGF) and EGF receptor. Current protocols in cell biology / editorial board, Juan S Bonifacino [et al]. 2010;Chapter 15:Unit 15 4. . 20235100 10.1002/0471143030.cb1514s46 PMC2878126

[49] Schmidt MH, Dikic I. Assays to monitor degradation of the EGF receptor. Methods Mol Biol. 2006;327:131–8. . 16780217 10.1385/1-59745-012-X:131

[50] Roepstorff K, Grandal MV, Henriksen L, Knudsen SL, Lerdrup M, Grovdal L, et al Differential effects of EGFR ligands on endocytic sorting of the receptor. Traffic. 2009;10(8):1115–27. Epub 2009/06/18. 10.1111/j.1600-0854.2009.00943.x 19531065 PMC2723868

[51] Shtiegman K, Kochupurakkal BS, Zwang Y, Pines G, Starr A, Vexler A, et al Defective ubiquitinylation of EGFR mutants of lung cancer confers prolonged signaling. Oncogene. 2007;26(49):6968–78. Epub 2007/05/09. 10.1038/sj.onc.1210503 . 17486068

[52] Turvy DN, Blum JS. Biotin labeling and quantitation of cell-surface proteins. Curr Protoc Immunol. 2001;Chapter 18:Unit 18 7. Epub 2008/04/25. 10.1002/0471142735.im1807s36 . 18432749

[53] van Weert AW, Geuze HJ, Groothuis B, Stoorvogel W. Primaquine interferes with membrane recycling from endosomes to the plasma membrane through a direct interaction with endosomes which does not involve neutralisation of endosomal pH nor osmotic swelling of endosomes. Eur J Cell Biol. 2000;79(6):394–9. Epub 2000/08/06. . 10928454 10.1078/0171-9335-00062

[54] Wiley HS, Burke PM. Regulation of receptor tyrosine kinase signaling by endocytic trafficking. Traffic. 2001;2(1):12–8. Epub 2001/02/24. . 11208164 10.1034/j.1600-0854.2001.020103.x

[55] Wong RW, Guillaud L. The role of epidermal growth factor and its receptors in mammalian CNS. Cytokine Growth Factor Rev. 2004;15(2–3):147–56. Epub 2004/04/28. 10.1016/j.cytogfr.2004.01.004 . 15110798

[56] Yarden Y, Sliwkowski MX. Untangling the ErbB signalling network. Nat Rev Mol Cell Biol. 2001;2(2):127–37. 10.1038/35052073 . 11252954

[57] Sigismund S, Confalonieri S, Ciliberto A, Polo S, Scita G, Di Fiore PP. Endocytosis and signaling: cell logistics shape the eukaryotic cell plan. Physiological reviews. 2012;92(1):273–366. Epub 2012/02/03. 10.1152/physrev.00005.2011 . 22298658 PMC5614474

[58] Platta HW, Stenmark H. Endocytosis and signaling. Curr Opin Cell Biol. 2011;23(4):393–403. Epub 2011/04/09. 10.1016/j.ceb.2011.03.008 . 21474295

[59] Grant BD, Donaldson JG. Pathways and mechanisms of endocytic recycling. Nat Rev Mol Cell Biol. 2009;10(9):597–608. Epub 2009/08/22. 10.1038/nrm2755 19696797 PMC3038567

[60] Maxfield FR, McGraw TE. Endocytic recycling. Nat Rev Mol Cell Biol. 2004;5(2):121–32. Epub 2004/03/26. 10.1038/nrm1315 . 15040445

[61] Sorkin A, von Zastrow M. Endocytosis and signalling: intertwining molecular networks. Nat Rev Mol Cell Biol. 2009;10(9):609–22. Epub 2009/08/22. 10.1038/nrm2748 19696798 PMC2895425

[62] Scita G, Di Fiore PP. The endocytic matrix. Nature. 2010;463(7280):464–73. Epub 2010/01/30. 10.1038/nature08910 . 20110990

[63] Dikic I. Mechanisms controlling EGF receptor endocytosis and degradation. Biochem Soc Trans. 2003;31(Pt 6):1178–81. Epub 2003/12/04. . 14641021 10.1042/bst0311178

[64] Eden ER, Huang F, Sorkin A, Futter CE. The role of EGF receptor ubiquitination in regulating its intracellular traffic. Traffic. 2012;13(2):329–37. 10.1111/j.1600-0854.2011.01305.x 22017370 PMC3261333

[65] Gruenberg J, Stenmark H. The biogenesis of multivesicular endosomes. Nat Rev Mol Cell Biol. 2004;5(4):317–23. 10.1038/nrm1360 . 15071556

[66] Qualmann B, Mellor H. Regulation of endocytic traffic by Rho GTPases. Biochem J. 2003;371(Pt 2):233–41. . 12564953 10.1042/BJ20030139 PMC1223314

[67] Ridley AJ. Rho GTPases and actin dynamics in membrane protrusions and vesicle trafficking. Trends Cell Biol. 2006;16(10):522–9. Epub 2006/09/05. 10.1016/j.tcb.2006.08.006 . 16949823

[68] Zhao ZS, Manser E, Loo TH, Lim L. Coupling of PAK-interacting exchange factor PIX to GIT1 promotes focal complex disassembly. Mol Cell Biol. 2000;20(17):6354–63. . 10938112 10.1128/mcb.20.17.6354-6363.2000 PMC86110

[69] Hoefen RJ, Berk BC. The multifunctional GIT family of proteins. J Cell Sci. 2006;119(Pt 8):1469–75. Epub 2006/04/07. 10.1242/jcs.02925 . 16598076

[70] D'Souza-Schorey C, Chavrier P. ARF proteins: roles in membrane traffic and beyond. Nat Rev Mol Cell Biol. 2006;7(5):347–58. Epub 2006/04/25. 10.1038/nrm1910 . 16633337

[71] Vitale N, Patton WA, Moss J, Vaughan M, Lefkowitz RJ, Premont RT. GIT proteins, A novel family of phosphatidylinositol 3,4, 5-trisphosphate-stimulated GTPase-activating proteins for ARF6. J Biol Chem. 2000;275(18):13901–6. Epub 2000/05/02. . 10788515 10.1074/jbc.275.18.13901

[72] Premont RT, Claing A, Vitale N, Perry SJ, Lefkowitz RJ. The GIT family of ADP-ribosylation factor GTPase-activating proteins. Functional diversity of GIT2 through alternative splicing. J Biol Chem. 2000;275(29):22373–80. Epub 2000/07/18. . 10896954 10.1074/jbc.275.29.22373

[73] Donaldson JG, Jackson CL. ARF family G proteins and their regulators: roles in membrane transport, development and disease. Nat Rev Mol Cell Biol. 2011;12(6):362–75. Epub 2011/05/19. 10.1038/nrm3117 21587297 PMC3245550

[74] Frank SR, Hansen SH. The PIX-GIT complex: a G protein signaling cassette in control of cell shape. Semin Cell Biol. 2008;19(3):234–44. Epub 2008/02/27. 10.1016/j.semcdb.2008.01.002 18299239 PMC2394276

[75] Di Cesare A, Paris S, Albertinazzi C, Dariozzi S, Andersen J, Mann M, et al p95-APP1 links membrane transport to Rac-mediated reorganization of actin. Nat Cell Biol. 2000;2(8):521–30. . 10934473 10.1038/35019561

[76] Matafora V, Paris S, Dariozzi S, de Curtis I. Molecular mechanisms regulating the subcellular localization of p95-APP1 between the endosomal recycling compartment and sites of actin organization at the cell surface. J Cell Sci. 2001;114(Pt 24):4509–20. . 11792816 10.1242/jcs.114.24.4509

[77] Valdes JL, Tang J, McDermott MI, Kuo JC, Zimmerman SP, Wincovitch SM, et al Sorting nexin 27 protein regulates trafficking of a p21-activated kinase (PAK) interacting exchange factor (beta-Pix)-G protein-coupled receptor kinase interacting protein (GIT) complex via a PDZ domain interaction. J Biol Chem. 2011;286(45):39403–16. Epub 2011/09/20. 10.1074/jbc.M111.260802 21926430 PMC3234764

[78] Prelich G. Gene overexpression: uses, mechanisms, and interpretation. Genetics. 2012;190(3):841–54. 10.1534/genetics.111.136911 22419077 PMC3296252

[79] Sorkin A. Endocytosis and intracellular sorting of receptor tyrosine kinases. Front Biosci. 1998;3:d729–38. Epub 1998/07/22. . 9671598 10.2741/A316

[80] Bitler BG, Goverdhan A, Schroeder JA. MUC1 regulates nuclear localization and function of the epidermal growth factor receptor. J Cell Sci. 2010;123(Pt 10):1716–23. Epub 2010/04/22. 10.1242/jcs.062661 20406885 PMC2864713

[81] Yarden Y. The EGFR family and its ligands in human cancer. signalling mechanisms and therapeutic opportunities. Eur J Cancer. 2001;37 Suppl 4:S3–8. Epub 2001/10/13. . 11597398 10.1016/s0959-8049(01)00230-1

[82] Blume-Jensen P, Hunter T. Oncogenic kinase signalling. Nature. 2001;411(6835):355–65. Epub 2001/05/18. 10.1038/35077225 . 11357143

[83] Roepstorff K, Grovdal L, Grandal M, Lerdrup M, van Deurs B. Endocytic downregulation of ErbB receptors: mechanisms and relevance in cancer. Histochem Cell Biol. 2008;129(5):563–78. Epub 2008/02/22. 10.1007/s00418-008-0401-3 18288481 PMC2323030

[84] Grovdal LM, Johannessen LE, Rodland MS, Madshus IH, Stang E. Dysregulation of Ack1 inhibits down-regulation of the EGF receptor. Exp Cell Res. 2008;314(6):1292–300. Epub 2008/02/12. 10.1016/j.yexcr.2007.12.017 . 18262180

[85] Yokota T, Kouno J, Adachi K, Takahashi H, Teramoto A, Matsumoto K, et al Identification of histological markers for malignant glioma by genome-wide expression analysis: dynein, alpha-PIX and sorcin. Acta Neuropathol. 2006;111(1):29–38. Epub 2005/12/02. 10.1007/s00401-005-1085-6 . 16320026

[86] Daniels RH, Zenke FT, Bokoch GM. alphaPix stimulates p21-activated kinase activity through exchange factor-dependent and—independent mechanisms. J Biol Chem. 1999;274(10):6047–50. Epub 1999/02/26. . 10037684 10.1074/jbc.274.10.6047

[87] Yoshii S, Tanaka M, Otsuki Y, Fujiyama T, Kataoka H, Arai H, et al Involvement of alpha-PAK-interacting exchange factor in the PAK1-c-Jun NH(2)-terminal kinase 1 activation and apoptosis induced by benzo[a]pyrene. Mol Cell Biol. 2001;21(20):6796–807. Epub 2001/09/21. 10.1128/MCB.21.20.6796-6807.2001 11564864 PMC99857

