# Supplementary figures and images for: αPIX Is a Trafficking Regulator that Balances Recycling and Degradation of the Epidermal Growth Factor Receptor
Source: PLoS One. 2015 Jul 15;10(7):e0132737. doi: 10.1371/journal.pone.0132737 (PMC4503440; doi:10.1371/journal.pone.0132737)

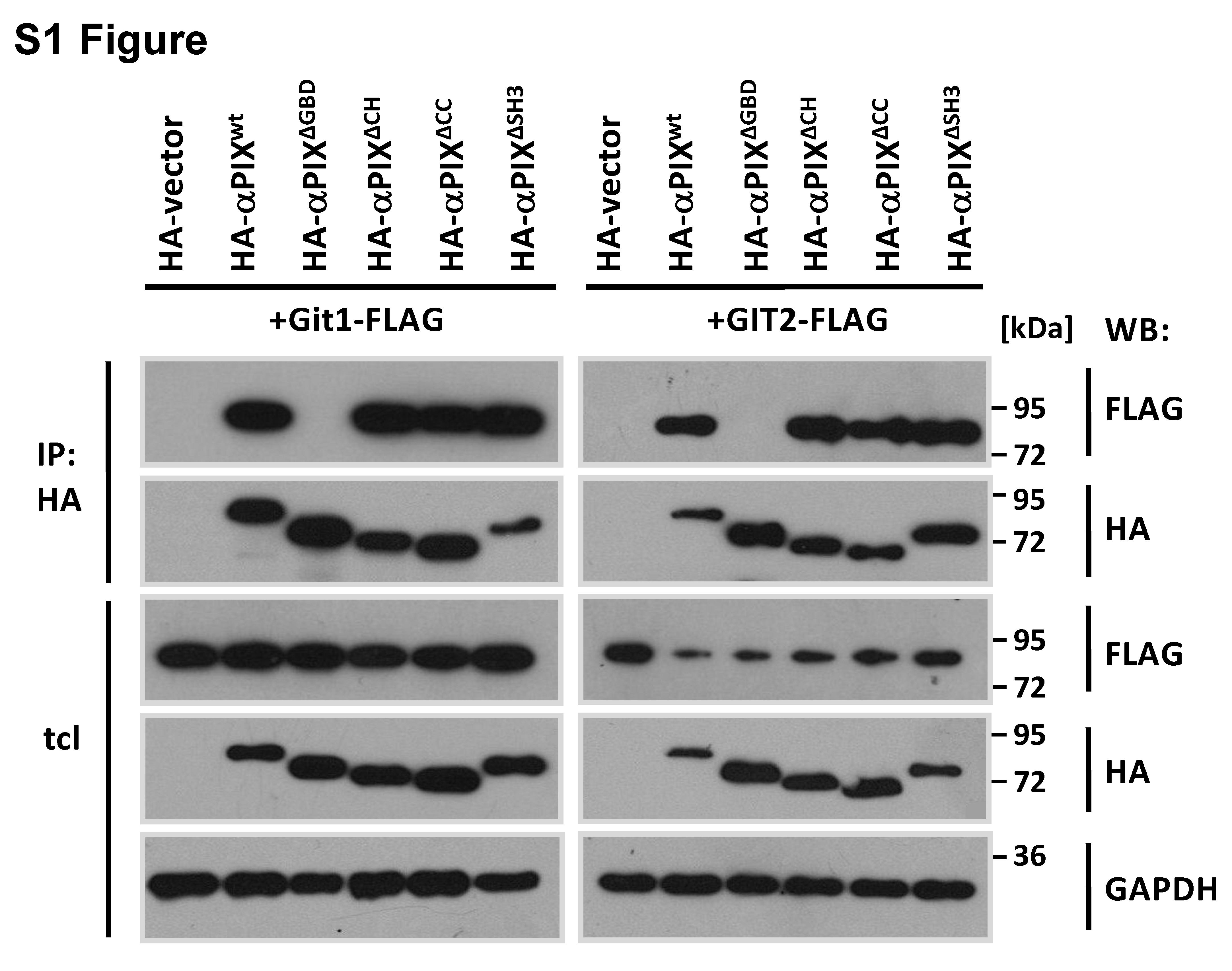

Supplement: S1 Fig — COS-7 cells were transiently co-transfected with the indicated expression constructs. For control purpose empty HA-vector was used. HA-tagged αPIX was immunoprecipitated from cell extracts by using anti-HA-conjugated agarose beads. After SDS-PAGE and western blotting, immunoprecipitates (IP) and total cell lysates (tcl) were probed with anti-HA and anti-FLAG antibodies. The HA-membrane was re-probed using anti-GAPDH antibodies to control for equal loading. Both, Flag-tagged rat Git1 and human GIT2 well co-precipitated with HA-αPIXwt (wild-type). In contrast, deletion of αPIX GBD (αPIXΔGBD) abolished co-immunoprecipitation of FLAG-Git1 and FLAG-GIT2 (top panel). All other tested αPIX deletion variants (αPIXΔCH, αPIXΔCC, αPIXΔSH3; please see Fig 1B) did not affect binding with FLAG-Git1 or FLAG-GIT2. (TIF) [file pone.0132737.s001.tif]

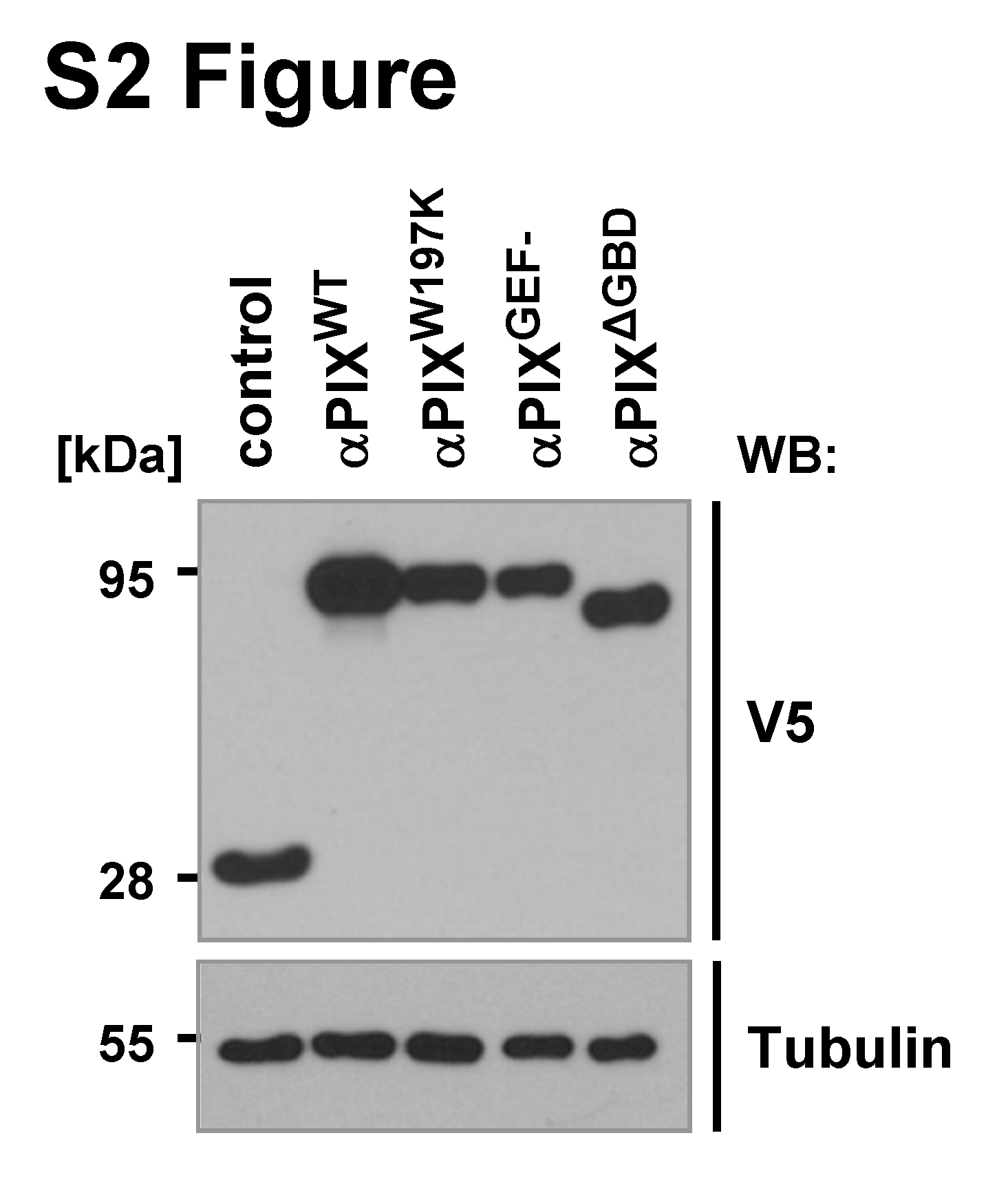

Supplement: S2 Fig — Cell lines stably overexpressing the indicated V5-tagged αPIX protein variants or V5-tagged CAT (control) were cultivated under basal growth conditions. Cell extracts were subjected to western blot analysis using anti-V5 antibodies. Tubulin served as a loading control. (TIF) [file pone.0132737.s002.tif]

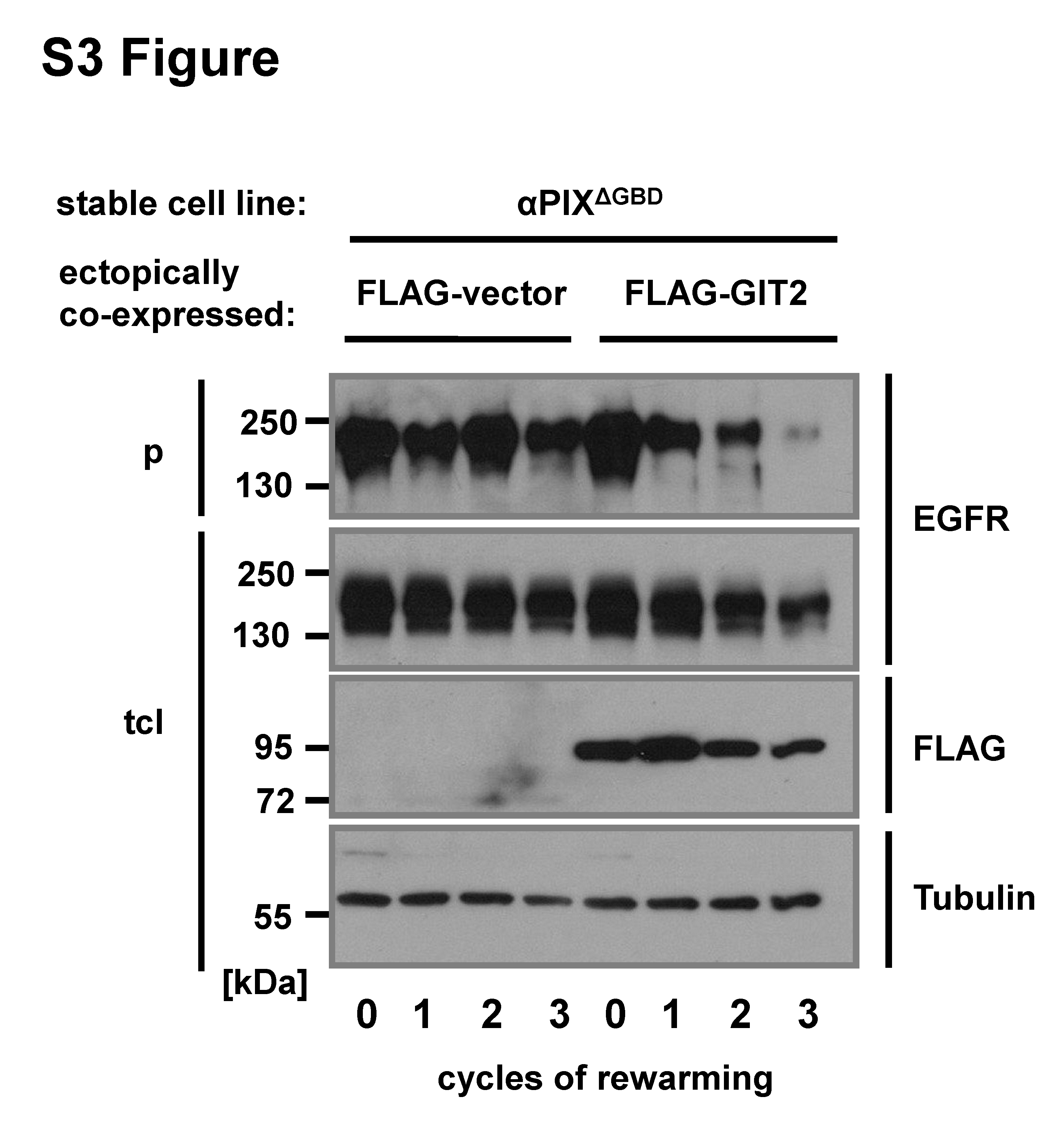

Supplement: S3 Fig — CHO cells stably expressing αPIXΔGBD were co-transfected with EGFR and GIT2 expression constructs followed by incubation in starvation medium supplemented with pepstatin A and leupeptin to inhibit lysosomal degradation. Surface proteins were biotinylated and cells were stimulated with 25 ng/ml EGF for 30 min at 37°C to induce EGF receptor trafficking. Subsequently, cells were transferred to 4°C and residual surface biotin was removed. Parallel cultures were subjected to 1, 2 or 3 cycles of 2 min rewarming at 37°C and de-biotinylation of recycled receptors. Intracellular biotinylated proteins were precipitated from cell extracts. Parallel cultures were harvested without rewarming/de-biotinylation (0 cycles). Total cell lysates (tcl) and precipitates (p) were subjected to SDS-PAGE and immunoblotting using anti-EGFR antibodies. Expression of FLAG-tagged GIT2 was verified by immunoblotting of tcl with anti-FLAG antibodies. Tubulin served as a loading control. We observed a reasonably constant intracellular EGFR pool over time (please see 1st, 2nd and 3rd cycle of rewarming) in cells expressing αPIXΔGBD but not GIT2 (FLAG-vector). In contrast the amount of intracellular EGFR gradually decreased in cells co-expressing FLAG-GIT2, suggesting that in the regulation of EGFR recycling GIT2 acts downstream of αPIX. (TIF) [file pone.0132737.s003.tif]

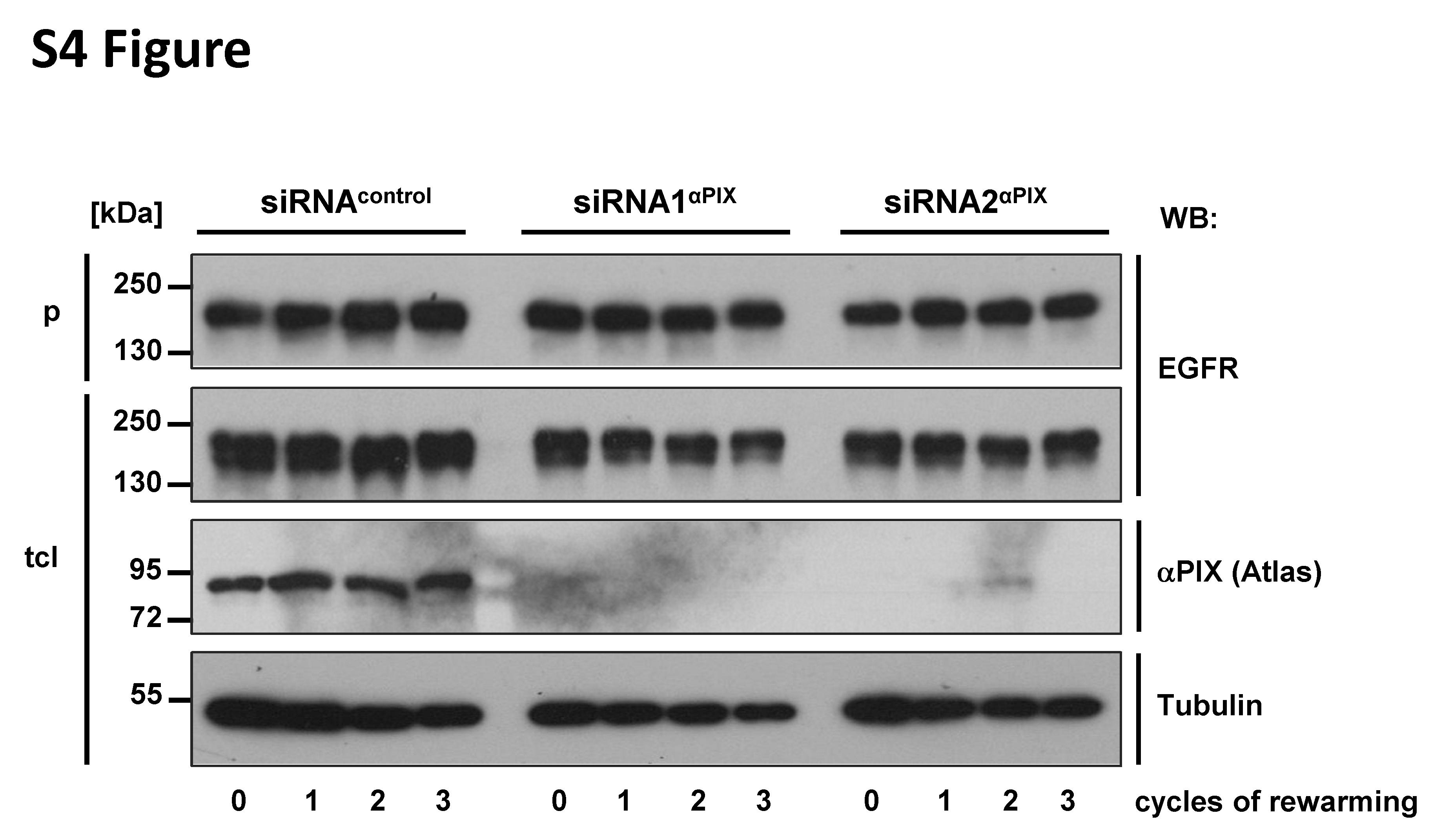

Supplement: S4 Fig — CHO-K1 cells were transfected with EGFR expression constructs and siRNA1αPIX, siRNA2αPIX or control siRNA (siRNAcontrol). 24h post transfection cells were incubated in starvation medium supplemented with pepstatin A and leupeptin for additional 24h to inhibit lysosomal degradation. Subsequently, surface proteins were biotinylated, and cells were treated with 25 ng/ml EGF for 30 min at 37°C to induce EGFR internalization. Cell surface-bound biotin was stripped off and cells were subjected to up to three cycles of rewarming to 37°C for 2 min and de-biotinylation of recycled receptors. Parallel cultures were harvested without rewarming/de-biotinylation (0 cycles). Intracellular biotinylated receptors were precipitated from cell extracts by streptavidin affinity gel. Total cell extracts (tcl) and precipitates (p) were analyzed by immunoblotting using anti-EGFR, anti-αPIX and anti-Tubulin antibodies. (TIF) [file pone.0132737.s004.tif]

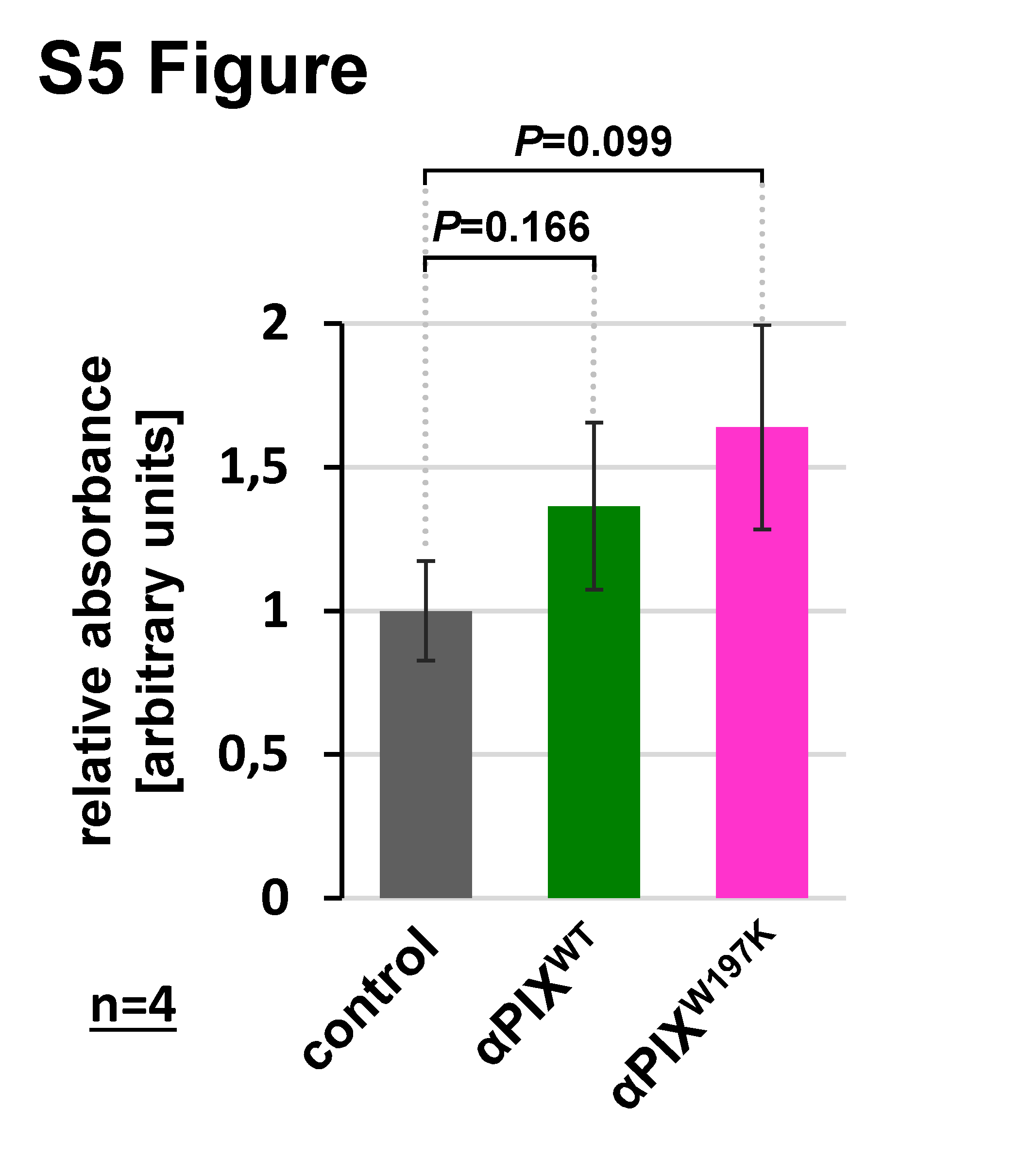

Supplement: S5 Fig — 12.500 CHO cells stably expressing CAT (control), αPIXWT or αPIXW197K were starved for 24h hours to synchronize the cell cycle. Subsequently, cells were stimulated with regular growth medium containing BrdU for 6h to induce proliferation and incorporation of BrdU during S-Phase. BrdU incorporation was measured photometrically. Graphs represent relative absorbance measured at 450 nm. For quantification the absorption of a cell-free well was subtracted and the mean value of CAT expressing control cells was used for normalization. Data represent the mean of four (n = 4) independent experiments ± sd. P values were calculated by paired Student’s t-test. (TIF) [file pone.0132737.s005.tif]
